# Catalytic/Protective Properties of Martian Minerals and Implications for Possible Origin of Life on Mars

**DOI:** 10.3390/life8040056

**Published:** 2018-11-05

**Authors:** Teresa Fornaro, Andrew Steele, John Robert Brucato

**Affiliations:** 1 Geophysical Laboratory of the Carnegie Institution for Science, 5251 Broad Branch Rd. NW, Washington, DC 20015, USA; asteele@carnegiescience.edu; 2 INAF-Astrophysical Observatory of Arcetri, L.go E. Fermi 5, 50125 Firenze, Italy; jbrucato@arcetri.astro.it

**Keywords:** Mars, minerals, biomarkers, catalysis, preservation, ionizing radiations

## Abstract

Minerals might have played critical roles for the origin and evolution of possible life forms on Mars. The study of the interactions between the “building blocks of life” and minerals relevant to Mars mineralogy under conditions mimicking the harsh Martian environment may provide key insight into possible prebiotic processes. Therefore, this contribution aims at reviewing the most important investigations carried out so far about the catalytic/protective properties of Martian minerals toward molecular biosignatures under Martian-like conditions. Overall, it turns out that the fate of molecular biosignatures on Mars depends on a delicate balance between multiple preservation and degradation mechanisms, often regulated by minerals, which may take place simultaneously. Such a complexity requires more efforts in simulating realistically the Martian environment in order to better inspect plausible prebiotic pathways and shed light on the nature of the organic compounds detected both in meteorites and on the surface of Mars through *in situ* analysis.

## 1. Introduction

The past and current exploration missions of the planet, Mars, have by now ascertained its past habitability [1,2], i.e., Mars possessed the geochemical complexity necessary for life to be maintained if present. The presence of mineral phases, like phyllosilicates, sulfates, and opals [3,4,5,6], tell us a story of aqueous alteration processes occurred on early Mars. Moreover, the great variety of oxidation states of the Martian mineral deposits points to intense redox chemistry [7]. Overall, the geochemical diversity observed on Mars suggests that this planet supported multiple habitable environments during its first billion years. Massive ancient sea-floor hydrothermal deposits have been postulated within the Eridania basin on southern Mars, which if confirmed would be evidence of a deep, long-lived sea and a deep-sea hydrothermal environment existing 3.7 billion years ago [8]. Observations from the NASA 2001 Mars Odyssey and the European Space Agency (ESA) Mars Express spacecraft revealed a current water content of the Martian regolith up to 2–15 wt% in the equatorial region [9,10], but the very low surface temperatures of Mars, ranging from 145 K during the polar night to 300 K on the equator at midday at the closest point in Mars’ orbit around the Sun [11], cause the formation of a several kilometers thick cryosphere where water is frozen. Nevertheless, recent results from the Mars Advanced Radar for Subsurface and Ionosphere Sounding (MARSIS) instrument on board the ESA Mars Express spacecraft point to a present-day subglacial salty lake of liquid water 20 km across, located 1.5 km beneath the ice at Planum Australe in correspondence with Mars’ South Pole [12]. This new discovery suggests that liquid water has had a long and significant impact on Mars.

Mars appears to meet also all the other requirements postulated for the origin of life and known for its sustenance, i.e., the availability of energy sources for driving chemical processes, catalysts, and nutrients [13]. Possible energy sources are radiations, geothermal heat, and a broad coupled redox chemistry. The chemical elements–carbon, hydrogen, nitrogen, oxygen, phosphorus, and sulfur–have all been detected on Mars. Carbon is found as gaseous carbon dioxide in the atmosphere, carbon dioxide ice, organic carbon, and carbonate minerals [14,15,16,17]. Complex refractory organic carbon has been discovered inside meteorites dating from 4.0 billion to 130 million years old as well as in the 3.2 billion year old Gale crater on Mars [18]. Therefore, complex organic molecules have been present and possibly available for most of Mars’ history. Hydrogen is currently present in hydrated minerals, as well as in water ice, organics, vapor, and liquid water, as recently detected in correspondence with the South Pole [12], and there is ample evidence of a much higher abundance of water on Mars in the past as already mentioned. Nitrogen constitutes only 2.7% of the Martian atmosphere and, in general, it is scarce on Mars, likely due to its high volatility and the low reactivity of molecular nitrogen, which makes very difficult reactions with other chemicals to form new compounds. However, nitrates have been detected on Mars and some nitrogen-bearing organic and inorganic compounds have been found in Martian meteorites [19,20,21,22]. Oxygen is an important element of minerals, like oxides, silicates, and sulfates, constituting the oxidized Martian crust. It can be found in water, in the carbon dioxide that dominates the Martian atmosphere, as well as being present as carbonyl and carboxylate groups in organic phases identified in meteorites. Phosphorus is also present on Mars in minerals, like apatite, which is a hydrous phase identified in all Martian meteorites recognized so far. The apatite content of Martian meteorites have been used to quantify the presence of water in the Martian mantle, estimated as 100 part per million (ppm), and revealed a close association between magmatic and hydrothermal activity on Mars [23]. Interestingly, Martian apatite has shown to be rich in chlorine, which determines higher solubility in aqueous solutions with respect to the typical fluorine-rich apatite of terrestrial basalts and, hence, greater availability of phosphate for prebiotic processes [24,25]. Chlorine has also been reported as being bound to carbon in the detection of chloromethane and benzochlorine species by the Sample Analysis at Mars (SAM) instrument on board the NASA Mars Science Laboratory’s (MSL) Curiosity rover [26]. Sulfur is present in minerals, like sulfates and sulfides, in the Martian atmosphere [27], as well as in organic compounds both in meteorites and on Mars (dimethylsulfide, methanthiol, and a range of thiophenes and derivatives) [18]. Other metals with central roles in terrestrial biology, like sodium, potassium, magnesium, and calcium, as well as transition metals, can be also found in Martian rocks (see Section 2).

The most interesting question at this point is: Did these known habitable environments on Mars lead to the emergence of life? If yes, assuming the emergence and evolution of a similar biochemistry on both early Earth and Mars, then we would expect that, in the absence of intense resurfacing processes on Mars, the exploration of its pristine habitable locations might shed light into the early life forms on Earth, whose biosignatures have been almost completely cancelled from our geological record due to metamorphism, overprinting by younger organisms, and weathering [28,29].

Important clues for addressing such issues can be found in the organic material detected so far in the Martian meteorites [21,30] and directly on the surface of Mars through *in situ* exploration [18,26,31]. It has been verified that the nature of the organic inventory found in the Martian meteorites is abiotic. Beyond the exogenous carbonaceous material delivered on Mars by meteorites [32,33] and interplanetary dust particles [34], the organic matter detected in the Martian meteorites derives also from a variety of endogenous igneous and secondary processes that likely occurred for most of the geological history of the planet [21,30,35,36].

Ancient organic molecules have also been recently discovered *in situ* by the Curiosity rover in the top five centimeters of the Martian regolith and within drilled mudstones, despite the current harsh conditions of the surface of Mars [18,21,26,31]. This discovery bodes well for the preservation of organics in more protected environments within the subsurface. Such environments will be explored by the upcoming ESA ExoMars 2020 mission [37] using a rover equipped with a drill capable of collecting samples up to a depth of two meters, where the radiation conditions are likely less extreme.

Understanding the origin of the organic molecules detected on Mars is not trivial because of possible transformations occurring inside the instrument used for their detection. Specifically, the Curiosity rover has used the SAM instrument [38], which can heat up to 875 °C the samples collected from the Martian regolith and monitor the volatiles released from the samples using a mass spectrometer. Firstly, it observed small organic molecules, such as chlorobenzene and C_2_ to C_4_ dichloroalkanes, coming off as detectable gases at temperatures below 400 °C [26,31,39]. Subsequently, it has been possible to detect also fragments of refractory material heating above 500 °C, which indicates the presence of much larger organic macromolecules originally present in the Martian samples [18]. This more complex material constitutes the dominant component of the total organic content of the samples analyzed. Such a high level, along with the observation that the overall geochemistry of the regolith at Gale crater is quite similar to that of other sites on Mars, like Gusev crater and Meridiani Planum, corroborate the hypothesis of a wider distribution of this kind of material on Mars at a global scale, which has definitely been one of the astrobiologically most relevant news by MSL. However, the organic compounds detected by SAM are generally only fragments deriving from the decomposition by high temperature pyrolysis of the original material, which has likely been subjected to additional complex chemical phenomena, such as chlorination, sulfurization, cyclization, and condensation, caused by the interaction with perchlorates/oxychlorine, sulfur dioxide, or other inorganic materials present in the Martian samples [18,26,31,39,40,41]. Thus, based on SAM analyses alone, it is not possible to recognize the nature of the original organic matter and distinguish among exogenous abiotic organics delivered by meteorites and interplanetary dust particles, or abiotic material formed through endogenous processes on Mars, or even ancient biotic material formed as consequence of life activity on Mars. Nevertheless, the discovery of these molecules in SAM data was due to work on a range of organics discovered in Martian meteorites and there is a direct similarity in the concentration and molecular speciation seen between the meteorites and SAM data [42]. Undoubtedly, some transformation has taken place during SAM analysis, but this similarity lends credence to the hypothesis that the organics are from an indigenous Martian abiotic pool. It is noteworthy that the organic molecules analyzed both in meteorites and by SAM represent those released at temperatures greater than ~600 °C and therefore represent a refractory pool. More labile sub ~600 °C organic species (other than chloromethane) must be present on Mars, but are overprinted by terrestrial contamination in the Martian meteorites, and by background in the SAM instrument [26,31]. It is these organics that represent the forefront of research into the presence or absence of life on Mars. In this temperature range, falls the nucleic and amino acids, phosphate bearing energy molecules, such as ATP, and their diagenetic products.

Beyond the transformation that occurred inside the SAM instrument, the original material might have been subjected to previous processing acting over geological time throughout Mars’ history, caused by several factors that may include: (i) Deposition and subsequent diagenesis in lake sediments from water, heat, and pressure; (ii) physical weathering by dissolution, water, and wind agitation and fragmentation, particle collisions, abrasion, glacial processes, volcanism, and impact shocks; and (iii) chemical weathering through irradiation and oxidation degradation [43]. The most dramatic degradation should have occurred during the most recent Amazonian period, due to the shutting down of the planet geodynamo and the erosion of the atmosphere by the solar wind, which resulted in high irradiation at the surface by galactic cosmic rays, energetic solar protons, and ultraviolet (UV) photons [44,45,46,47,48,49,50,51], along with the formation of strong oxidants in the soil driven by photochemical processes [52,53,54]. Currently, Mars is not very geologically active, so the primary mechanism of physical weathering is aeolian erosion caused by the collision of particles moved by the wind. The physical weathering causes the exposition of fresh material to radiation, which is then subjected to chemical weathering. Another current significant degradation agent is constituted by the presence of oxidants in the regolith, such as perchlorates, that have been detected by both orbital remote sensing techniques and instruments on board the NASA Phoenix Lander and the Curiosity rover [52,53,54,55]. SAM measurements suggest a concentration of chlorate/perchlorate in the range 0.05–1.05 wt% [56], although oxidants should be absent yet at a depth of 2–4 m in the subsurface, according to a model developed by Patel et al. [46].

Minerals played a crucial role in most of the above-mentioned processes experienced by organic molecules on Mars, influencing their chemical evolution, with implications in the possible origin of life on this planet [57]. Indeed, mineral surfaces provide a solid support to concentrate organic molecules from dilute aqueous environments through chemisorption or physisorption [58,59], and the structure of some minerals may act as templates for prebiotic reactions [60]. The molecule-mineral interactions may favor molecular self-organization and catalyze reactions toward more complex species [59,61,62]. Chemical reactions catalysed by minerals include [63,64,65,66]: The Strecker synthesis, in which ammonia, hydrogen cyanide, and aldehydes react to produce amino acids and related products; the Fischer-Tropsch process, which is a high-temperature reaction of carbon monoxide and hydrogen to give hydrocarbons; FeS-driven organic synthesis; water-rock reactions, like the serpentinization process, where water oxides iron(II) present in olivine to iron(III) producing molecular hydrogen, which in turn may react with carbon dioxide forming hydrocarbons and reduce nitrogen to ammonia; or photo-catalytic processes, like the transformation of formamide in the presence of a mixture of TiO_2_ and ZnO metal oxides that produce carboxylic acids, amino acids, and nucleic acid bases. Furthermore, some minerals may play a role in enhancing degradation of specific organic molecules, while other minerals, on the contrary, favor preservation [57,67]. For these reasons, it is fundamental to investigate the roles of the Martian minerals in the physico-chemical processes that might have occurred on the planet in the past or that may currently take place, in order to validate possible scenarios about the nature of the organic compounds detected so far on Mars. In particular, relevant aspects that we will explore in this contribution concern the possible roles of minerals in degradation/preservation processes relevant to the origin and evolution of life on Mars. Firstly, we will provide a brief summary of the main classes of minerals identified on Mars (Section 2) and the organic compounds detected so far both through *in situ* exploration of Mars and Earth-based laboratory analyses of Martian meteorites (Section 3). Furthermore, we will discuss the catalytic/protective properties of minerals relevant to Mars’ mineralogy under Martian-like conditions, with a specific focus on their interactions with molecular biosignatures, the so-called biomarkers (Section 4). Finally, we will provide a summary of the results and take-home messages in Section 5, and the implications for future Martian missions in Section 6.

## 2. Martian Minerals

The landed and orbital data sets gathered so far have provided us with a wide mapping of Mars mineralogy [68], showing that the most abundant minerals on Mars are silicates, oxides, and sulfides, followed by carbonates, sulfates, chlorides, and perchlorates, as summarized in Table 1.

Among the Martian minerals, the “water-signature” ones draw interest for potential prebiotic processes and preservation of biostructures. Specifically, phyllosilicates, sulfates, and opals have been widely identified through remote sensing and *in situ* measurements [6,69,70,71,72], and represent striking evidence of a past aqueous activity on Mars. The characterization of these minerals on Mars and their distribution gives key information about near-surface aqueous alteration processes on ancient Mars.

Phyllosilicates’ formation requires abundant surface liquid water and/or hydrothermal activity, and a chemical environment alkaline to neutral. This occurred during the most ancient and flourishing geological era in Mars’ history, the so called Noachian (~4.1 to 3.7 billion years ago), as a consequence of weathering of the basaltic crust by liquid water. Therefore, phyllosilicates can be found in the oldest mineral deposits on Mars.

A subgroup of phyllosilicates particularly interesting in the prebiotic context is clay minerals due to their large surface area and optimal interlayer sites for the concentration and preservation of organic compounds when rapidly deposited under reducing chemical conditions [73]. These minerals have been detected at several locations on Mars [4,74,75,76,77,78,79,80,81,82], including Echus Chasma, Mawrth Vallis, Eridania basin, the Memnonia quadrangle, the Elysium quadrangle, Nili Fossae, and the large Argyre Planitia area. Most of these clays derive from surface alteration, indicating that there was an active hydrosphere and atmosphere on Mars, whereas only a small part comes from subsurface alteration. A high abundance of clay minerals, especially ferrian saponite, was also discovered by the Curiosity rover in fluvio-lacustrine sedimentary rocks at Gale crater dating back to ~3.5 billion years ago [1,81,83]. During its traverse of the Gale crater, the rover detected at different locations a diversity of clay minerals, such as a mixture of aluminium-rich dioctahedral and magnesium-rich trioctahedral smectites or micas and pyrophyllite-talc, with evidence of redox stratification and pH variations [84,85]. It also observed changes in minerals that are highly sensitive to the environment, specifically an overall reduction in the quantity of reactive mafic minerals, like pyroxene and olivine; a transition from magnetite to hematite as the main iron oxide; and increasing abundances of calcium sulfates that indicate near-surface evaporative processes and neutral or mildly alkaline conditions of the soil. The features of the sediments together with these mineralogical trends denote a very dynamic aqueous environment in the lake characterized by shallowing and episodic desiccation processes, and indicate that near-surface aqueous alteration continued into the Early Hesperian (Bristow at al. 2018 [86] and references therein). These kinds of processes might have played a fundamental role in possible biogeochemical cycles developed on early Mars. The great variety of chemicals identified at the Gale crater, characterized by different oxidation degrees, might have provided a sort of chemoautotrophic energy gradient capable of supporting life, based on the redox couples, Fe^3+^/^2+^, Mn^4+^/^2+^, and S^6+^/^2−^.

The NASA Mars Exploration Rovers (MER) Spirit and Opportunity also found strong evidence of liquid water on the surface, but in places on the planet that were highly acidic and salty. Combining the observations of the Compact Reconnaissance Imaging Spectrometer for Mars (CRISM) onboard the NASA Mars Reconnaissance Orbiter (MRO) and the ones from the Opportunity rover, it was possible to detect clay minerals in various locations along Endeavour’s western rim, including the smectites montmorillonite and nontronite in Marathon Valley, which showed to possess still a basaltic composition with relatively low water to rock ratios. Thus, their formation should have been associated with a low amount of water and likely occurred under mildly acidic conditions on early Mars, which is consistent with the lack of extensive formation of carbonate deposits [72].

Interestingly, lots of evaporites (chloride salts) have been detected to be spread all over Mars, likely formed after the deposition of phyllosilicates, covering clay-bearing material especially in the southern highlands within Noachian-aged terrains [87,88,89]. These evaporites may form *in situ* in chemical environments that range from alkaline to acidic. Their formation may occur in a variety of geological settings from brine to fluvial valleys, and requires a limited amount of liquid water that can derive from surface runoff due to ice/snow melt, precipitation, and/or groundwater upwelling. Chlorides are commonly the uppermost mineral in a Martian stratigraphic column, and rarely show traces of erosion and degradation. It might be possible that such a chloride-rich upper layer aids preservation of biosignatures on Mars.

The second major class of hydrated minerals mapped by the ESA Mars Express OMEGA instrument and detected by the NASA rovers is sulfates [69,90,91], including magnesium sulfates (such as kieserite) and calcium sulfates (such as gypsum). They have been found in locations, like the Gale crater, Endeavor crater, and at the North and South Poles of Mars. These kinds of minerals formed during an acid wet phase due to volcanic outgassing of volatiles, including sulfur, from the evaporation of salty and sometimes acidic water.

The MRO has also discovered opaline deposits across large regions of Mars [70], including the large Martian canyon system called Valles Marineris and the Home Plate in the Gusev crater. Opalines are the youngest hydrated mineral ever detected on Mars and provide indications of the presence of liquid water as recently as two billion years ago. Opalines can be formed by aqueous alteration of materials created by volcanic activity or meteorite impact, or hydrothermal activity as interpreted in the case of the Gusev crater [92]. In some locations around dry river channels, opaline deposits have been found in association with iron sulfates, revealing that acidic water remained on the Martian surface for a long time [93].

Another primary class of minerals detected on Mars by *in situ* measurements of both Viking Landers (Chryse Planitia and Utopia Planitia in 1976), Mars Pathfinder (Ares Vallis in 1997), and Spirit and Opportunity rovers (in Gusev Crater and Meridiani Planum, respectively) is anhydrous ferric oxides, which are responsible for the typical red color of the Martian surface. These minerals formed in the most recent geological era beginning about 3.5 billion years ago through a slow superficial weathering process [69]. The most stable iron oxide in the current Martian conditions is hematite [94], whose spectral signature was detected by the infrared spectrometer on board the NASA Mars Global Surveyor (MGS) and 2001 Mars Odyssey spacecraft in orbit around Mars in several locations [95,96], like Terra Meridiani, Valles Marineris, Aureum Chaos, Columbia Hills, etc. *In situ* analyses at Terra Meridiani by the Opportunity rover showed a significant amount of hematite in the form of small spherules, nicknamed as “blueberries”, which are apparently concretions formed as a consequence of precipitation from iron-rich water [97,98,99,100]. Hematite can be considered another “water-signature” mineral, since it can be formed by hydrothermal processes [98] and palagonitisation (aqueous alteration at low temperature) of volcanic ashes or glass [101], and be collected in layers at the bottom of lakes, springs, or other standing water environments. Hematite can also form without the intervention of water as a secondary mineral by meteoric weathering processes in the soil [102,103]. As previously mentioned, at Gale crater, the Curiosity rover found patterns of change in rock composition at higher, younger layers of Aeolis Mons, better known as Mount Sharp, observing that hematite replaced less-oxidized magnetite as the dominant iron oxide, which suggests warmer and more oxidizing conditions, or more interaction between the atmosphere and the sediments [104].

Regarding mafic minerals, OMEGA and previous instruments have revealed that pyroxene and olivine are still present at the surface in the older terrains included within sand dunes associated to ancient Noachian crustal rocks and early Hesperian volcanism [105,106]. However, much of the younger surface, particularly within the large lowlands of the northern hemisphere, does not exhibit mafic spectral signatures. The exposed original materials have been heavily chemically altered or covered by heavily altered dust [105,106,107].

Another significant component of the Martian regolith recently discovered by the Curiosity rover at Gale crater is amorphous material. Unfortunately, due to its low crystallinity, such a material is poorly characterized by the instruments available on board the Curiosity’s payload. However, the Chemistry and Mineralogy (CheMin) X-ray diffraction instrument has been able to provide some indications about individual phases within the amorphous material, like volcanic (or impact) glass, hisingerite (or silica + ferrihydrite), amorphous sulfates (or adsorbed SO_4_^2−^), and nanophase ferric oxides [86,108]. This material draws particular attention for the preservation of organic matter [109], and its in-depth characterization remains one of the challenges of the next missions.

## 3. Organics Detected on the Surface of Mars and in Martian Meteorites

Steele et al. have undertaken a comprehensive review of organics discovered in Martian meteorites [21]. To summarize this review, every class of Martian meteorites has been shown to contain a high temperature release (>600 °C) organic carbon inventory that is indigenous to the meteorite. There are variations among meteorites in the concentration of reduced carbon released, but there is no systematic trend associated with meteorite type. This high temperature reduced carbon inventory contain ~18 ± 26 ppm of reduced carbon with an average δ^13^C of ~−19.1 ± 4.5‰ (an anomalously high measurement of 52.8 ppm carbon in Zagami contributes to the large standard deviation in the reported carbon abundances in Martian rocks) [30,110]. Interestingly, the data collected by the SAM instrument on high temperature released organics (>500 °C) show an average of 11.15 ± 6.86 ppm across the Murray and Sheepbed mudstones [18]. The nature of the high temperature release organics on Mars show thiophenes, aromatics, aliphatics, and thiol derivatives. A similar distribution has been shown in organics released during the analysis of the Tissint meteorite [18]. Analysis of Tissint meteorite shows also the presence of aliphatic and aromatic nitrogen-containing organic molecules, such as pyrole [22], which instead have not been discovered in the Curiosity dataset.

The origin of the organics analyzed by Curiosity is the subject of some debate with two main sources: indigenous synthesis or meteoritic in-fall. However, the provenance of organics in Martian meteorites shows definitive evidence for organic synthesis in these rocks that reveals a similar organic distribution to that seen by Curiosity [30,111,112]. Beyond the carbonaceous material delivered by steady in-fall of interplanetary dust particles and carbonaceous meteorites, Martian meteorites show definitive evidence for abiotic organic synthesis occurring in these rocks through different mechanisms, including [21]: (1) impact-generated graphite (possibly diamond) in Tissint meteorite and magnetite/macromolecular carbon (MMC) clusters not contained within carbonate globules in ALH 84001 meteorite; (2) secondary hydrothermally generated MMC/graphite in ALH 84001 meteorite; (3) primary igneous reduced carbon; and (4) possible primary hydrothermally formed nitrogen-bearing organic compounds. The study of reduced carbon in Martian meteorites indicates that Mars has produced reduced carbon/organic carbon for most of its geologic history. Therefore, even though some transformation has undoubtedly taken place during SAM analysis, the most parsimonious explanation for the direct similarity in concentration and molecular speciation denoted between the analyses of Martian meteorites and SAM data is that the Curiosity analysis most likely sampled organics synthesized on Mars. More recently, Steele et al. [42] have shown a novel organic synthesis mechanism involving the electrochemical reduction of CO_2_ to organic material in Mars meteorites, which may constitute a plausible endogenous route for producing on Mars organic molecules that are the building blocks of life. Exploring the catalytic properties of Martian minerals under conditions reproducing Mars’ early environment appears fundamental to shed light on the possible prebiotic pathways that might have brought to the emergence of life on this planet.

## 4. Preservation of Biosignatures

Based on observations of terrestrial analog sites, it seems that sediments, evaporates, and hydrothermal systems feature the highest preservation potential for biosignatures [113].

On Earth, entombment of biosignatures within mineral matrices constitutes of one of the most efficient preservation mechanisms against degradation of both microorganisms and organic molecules. Entombment occurs through mineralization processes, including the ones driven by evaporation and precipitation, concentration of brine solutions by freezing, supersaturation due to cooling or changes in pressure, and diffusion-driven reactions leading to the formation of concretions. Another important mechanism for preservation is the enrichment inside mineral matrices as denoted in sedimentary rocks and phyllosilicate minerals, like clays. The reduced permeability characterizing fine-grained sediments and precipitates limits the exposure of entrapped organics to migrating fluids and gas. Sedimentation may enhance biosignature preservation by accumulating and burying biomass. Marine sediments on Earth have been found to protect organic molecules through their sorption into the mineral matrix of clays and metal oxides [114]. Specifically, iron oxides show a strong affinity for organics, forming stable complexes that can persist for thousands of years in anoxic sediments at depths of up to 5 m [115]. Sediments on Earth have shown to also preserve morphological biosignatures, such as trace fossils, wrinkle marks, stromatolites, and microbialites. However, the long-term preservation of terrestrial biosignatures in several paleo-environments on Earth depends on the persistence of the sedimentary material itself [116]. As analyzed in detail by Farmer and Des Marais [116], phosphates and silica, followed by clay-rich fine-grained sediments, carbonates, and metallic oxides, present the highest preservation potential, and terrestrial biosignatures can be affected only by deep burial and recrystallization during metamorphism or by dissolution. Metallic sulfides are more sensitive to oxidation. Halides and sulfate matrices are susceptible to dissolution. Ice appears to be the less effective protector since it is easily perishable by climatic warming.

**Figure 1:**
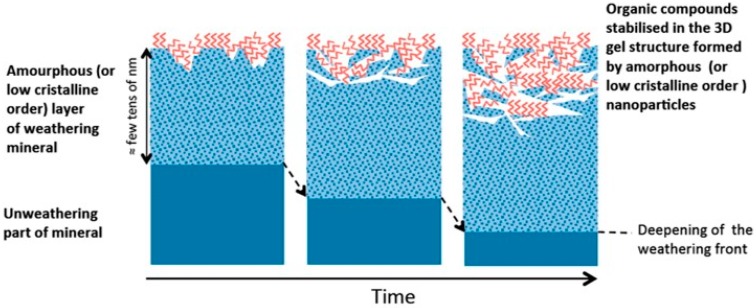
Model for the stabilization of organic compounds by amorphous material (Adapted with permission from Basile-Doelsch et al. 2015 [109]. Copyright 2015, American Chemical Society).

In regard to molecular biosignatures, molecular binding to minerals, like phyllosilicates [60,73,74] and Al-Fe oxyhydroxides [115], has shown to enhance preservation. Amorphous materials have also demonstrated some preservation potential that can be ascribed to the possibility to incorporate and protect molecules within their porous structure. An example is the study by Biondi at al. about the stability of RNA molecules adsorbed on opals. Results showed that the interaction with opal considerably stabilizes RNA against alkaline degradation with respect to the case of free molecules in aqueous solution at pH 9.5 [117]. Moreover, a preservation mechanism that may be particularly relevant for Mars is the one observed by amorphous materials derived from the weathering of surface rocks [109]. The weathering of volcanic rocks rapidly produces poorly crystallized nanometric-sized surface secondary phases (allophane type), consisting mainly of hydrolyzed Al, Si, and Fe, which are characterized by a large surface area (and, consequently, great affinity to organic compounds) and high surface reactivity. It has been observed that, in any soil, secondary nanosized phases newly formed by the weathering of surface minerals may give rise to nanosized organo-mineral complexes and stabilize organic compounds embedded into the 3D gel structure formed by the amorphous nanoparticles (Figure 1).

Phyllosilicate, iron oxyhydroxide, and amorphous materials are the dominant mineral species detected in all the mudstones investigated *in situ* by the Curiosity rover at Gale crater [84,85]. Moreover, the lower stratigraphy of Mount Sharp shows a transition from clay-bearing to sulfate-bearing strata [118]. Such a change likely occurred in the Late Noachian until the Hesperian due to the drop of the Martian atmospheric pressure and consequent increase in the amount of sulfur dioxide degassed from lavas [119]. The high abundance of thiophenic and total organic sulfur detected in Mojave and Confidence Hills samples drilled in the Murray formation mudstones suggests that sulfurization of organic materials prior to deposition and during early diagenesis is another potential preservation mechanism on Mars, which might have occurred during early diagenesis in the presence of reduced sulfur (HS^−^ or H_2_S) gas more than three billion years ago [18]. Furthermore, sulfur-bearing organics have also been observed in carbonaceous meteorites [120], and there is an indication of their presence in the Tissint Martian meteorite [18,42]. Natural vulcanization on Earth results in an enhanced refractory state for organic materials because sulfur is able to reduce reactive functional groups and add cross links between small unstable molecules, thereby converting them into recalcitrant macromolecules. In addition, sulfur may provide an additional oxidative sink for degradation reactions during diagenesis, so the iron sulfides detected in the Sheepbed mudstone [81] and suspected in the original Murray mudstone detritus [84,85] might have helped organic matter preservation. Interestingly, terrestrial sulfate minerals have shown to efficiently trap and preserve organic molecules within their structure [121,122]. Notable ability to preserve organic molecules has been observed also in terrestrial analog sites characterized by halite- and perchlorate-rich hypersaline subsurface deposits [123], which are widespread on Mars.

Given Mars’ mineralogy and lithology, it appears that a number of diverse environments exist on Mars where the likelihood of the preservation of biomarkers can be rather significant. In such locations, prebiotic processes might have taken place, leading to the emergence of a complex biochemistry.

In order to explore the mechanisms that might have or might not have led to the origin and evolution of life on Mars, it is fundamental to deepen the roles of Martian minerals in the multiple complex processes of preservation/degradation contributing to the final fate of possible biosignatures. These investigations would be key also to shedding light on the possible biogenicity of the organic compounds detected so far on Mars.

In general, preservation of biosignatures on Mars depends on numerous factors, such as the hydrogeological and atmospheric setting, the physico-chemical properties of mineral matrices, the kind of biosignature, etc. [43]. Minerals and chemical biosignatures can be affected by temperature and pressure changes, along with migrating fluids, which can modify the mineral phases, as well as dissolution and oxidation/reduction. Stable isotopic patterns can be altered by diagenesis, dissolution/recrystallization, and thermal processing. Organic compounds are easily degraded through: (i) Oxidation, which is a chemical reaction transforming reduced carbon structures into carbon dioxide and water by progressive introduction of oxygen; (ii) photolysis/radiolysis, which is a decomposition of the molecules induced by light/ionizing radiations; and (iii) thermal processing that may determine bond breakage and loss of molecular functional groups, as well as structural rearrangements towards higher thermodynamic stability [124].

In the following section, we will mainly focus on the role of minerals in the possible preservation/degradation mechanisms of molecular biosignatures, the so called biomarkers, under Martian conditions, considering the effects of oxidants and ionizing radiations. On Mars, thermal processing is not really an issue due to the low temperatures, which instead favor preservation. Thus, thermal processing will not be discussed in this contribution.

### 4.1. Oxidants on Mars

The failure of the Viking program in the late seventies to detect organic molecules on the surface of Mars was promptly attributed to the existence of powerful oxidizing agents in the regolith. Perchlorate ions (ClO_4_^−^) in salts in the regolith and hydrogen peroxide (H_2_O_2_) in the atmosphere have been identified as the main oxidants on Mars [125]. Their presence may have important astrobiological implications because oxidants can increase the reactivity of the soil, oxidize organic matter, and lower the freezing point of water, allowing for liquid brines to exist at the surface [126] or liquid water in the subsurface [12].

Perchlorates are generally highly soluble, so they can be found only in locations characterized by low water content. The Phoenix Lander was the first to detect calcium, magnesium, and sodium perchorates in the Martian regolith at a concentration of 0.4–0.6 wt% [53,127]. Further detection by the Curiosity rover at Gale crater at a concentration of 0.05–1.05 wt% [56] and in the Martian meteorite, EETA79001, with a concentration of 0.6 ± 0.1 ppm by mass [128], corroborated the hypothesis that they are widespread on Mars. The formation of perchlorates might have happened in the atmosphere, driven by UV radiation, in the presence of hydrochloric acid gas, emitted by past volcanism, released by aerosols present in Mars’ atmosphere, or through reactions between water ice and oxychlorine radicals produced by the atmospheric oxidation of chlorine (more details can be found in Lasne et al. [125], and references therein). However, mechanisms requiring atmospheric chlorine are insufficient to explain the concentrations measured on Mars [129]. Other mechanisms likely involve photochemical production by irradiation of chlorine-bearing regolith [130], or electrochemical processes during dust events [131]. The presence of perchlorates in the regolith would not affect the stability of organic compounds at the typical low temperatures on Mars due to the low reactivity of perchlorate at low temperatures, and the very slow oxidation kinetics expected for molecules, like amino acids, purines, and pyrimidines [132], while the thermal decomposition of perchlorates significantly disturbs the detection of organics in the Martian soil through *in situ* measurements based on pyrolysis [125]. However, ionizing radiations can decompose perchlorate even at low temperatures, producing several lower oxidation state oxychlorine species, like chlorate (ClO_3_^−^), hypochlorite (ClO^−^), and chlorine dioxide (ClO_2_), as well as molecular oxygen that remains trapped in the salt crystal [133], and atomic oxygen [134] or chlorite radicals (ClO_2_•) [135]. Such reactive species, in turn, may be responsible for the degradation of organic matter [133,136,137,138]. Interestingly, the SAM instrument indicated the presence of oxychlorine phases in Gale Crater, but the *CheMin* X-ray diffractometer was not able to detect them [139]. This observation can be explained if the oxychlorine in Gale Crater is poorly crystalline, or its concentration is below the 1 wt% detection limit of *CheMin*, which means that the concentration of oxychlorine species may vary across the planet. Unfortunately, only a few studies have been reported in the literature about the effect of irradiation on the stability of organic molecules in the presence of perchlorates [136,137], while many studies have tried to reproduce the pyrolysis results of the Viking, Phoenix, and MSL missions (see Lasne et al. [125], and references therein).

Hydrogen peroxide has been detected so far only in the atmosphere of Mars, at levels ranging from 18 to a maximum of 40 part per billion (ppb), with seasonal variations depending on the abundance of atmospheric water vapor and water ice clouds [140,141,142]. The formation of hydrogen peroxide in the atmosphere has been predicted considering photochemical processes [143] and electrochemical reactions during dust devils and storms [143,144,145]. Even though there is no direct detection of hydrogen peroxide in the regolith, its diffusion in the Martian subsurface has been theoretically predicted up to a maximum depth of a few centimeters, or hundreds of meters in the presence of impact gardening [146,147,148]. A plausible route for the production of hydrogen peroxide directly in the Martian regolith is based on water-mineral interactions, through dissociative chemisorption of water molecules onto the mineral surfaces followed by redox formation and the combination of hydroxyl radicals [149,150,151]. Furthermore, a recent study by Crandall et al. revealed a formation mechanism by which hydrogen peroxide and other potential oxidants can be generated via irradiation of perchlorate by cosmic rays [134]. The mix of iron, water, and hydrogen peroxide on Mars may result in a high oxidizing potential due to the production of reactive radical species through processes, like the Haber-Weiss cycle. Lasne et al. [125] describe such processes in detail, and propose a scheme of the oxidation state of the various layers that compose the Martian regolith, where the most oxidized superficial layer contains a superoxide ion (O_2_^−^•) and hydroxyl (OH•) radicals originating from the interaction between the regolith, the atmosphere, and UV radiation [55,152]. Plausible mechanisms include the photo-induced decomposition of hydrogen peroxide adsorbed on oxide minerals, and the photo-induced electron transfer from the surface of oxide minerals to water or oxygen molecules adsorbed on the surface as well as structural water molecules embedded into the crystal lattice. Transition metal oxides widely present on Mars feature small gaps between the valence and the conduction band, and can act as semiconductors by absorbing radiation and creating electron-hole pairs called excitons. Insulators, like feldspars and zeolites, may show a similar behavior [67] thanks to defects in their crystal structure, which change their electronic properties [152]. The excitons may recombine or diffuse inside the mineral grains and reach adsorbed molecules, promoting redox reactions. In the presence of molecular oxygen or water molecules adsorbed on the mineral surfaces, excitons may lead to the production of reactive oxygen species, like superoxide radical anions (O_2_^−^•), hydroxyl radicals (OH•), and hydroperoxyl radicals (HO_2_•), which, in turn, may degrade adsorbed organic molecules. The adsorption of oxygen and water onto mineral surfaces is quite plausible. Indeed, oxygen is present in the Martian atmosphere with an abundance of 0.13%, and models predict [152,153,154] the ubiquitous presence of a few monolayers of water on exposed Martian surfaces. In the case of minerals with high iron content, water adsorbed at the mineral surface can leach soluble species from the rock, leading to favorable conditions for Fenton and photo-Fenton reactions that may contribute to molecular degradation [155].

**Figure 2:**
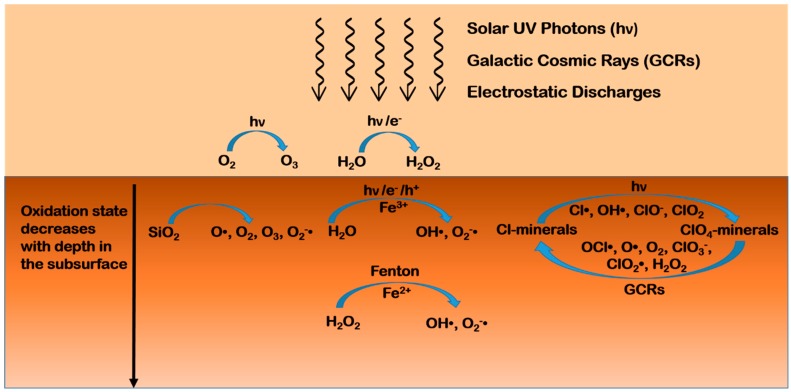
Simplified scheme of the oxidation environment of the Martian regolith.

The diffusion depth of hydrogen peroxide determines the thickness of the mildly oxidized layer, but the uncertainty about the hydrogen peroxide content in the regolith reflects on the results of the models, thus estimating an oxidant extinction depth between a few centimeters to hundreds of meters [146,147,152]. The penetration depth of perchlorates is also currently unknown. If their formation mechanisms include only UV radiation or electrostatic discharge in the atmosphere, and there is no replenishment in the subsurface, then perchlorates should concentrate mainly near the surface and their decomposition by cosmic rays should limit their diffusion at depths of no more than a few meters. A simplified scheme of the oxidation environment of the Martian regolith is depicted in Figure 2.

Given the spreading of oxidants on the surface and in the subsurface of Mars, it is fundamental to investigate their possible effects on organic matter under simulated Martian conditions. Mancinelli [156] and McDonald et al. [157] obtained interesting results by studying the stability of organic macromolecules, such as tholins and humic acid, subjected to an aqueous solution of hydrogen peroxide at different temperatures. Specifically, their data suggest that some organic macromolecules may be stable against oxidation on the Martian surface, at least in the polar regions, over the entire history of Mars. Dionysis et al. [158] evaluated the oxidation effect of dissolved hydrogen peroxide on the kinetics of formic acid decarboxylation in the presence of iron oxides, conducting a series of flow-through hydrothermal experiments at temperatures ranging from 80 to 150 °C and pressures of 172–241 bar. Their data revealed an increase in hydrogen peroxide decomposition in the presence of magnetite, which was likely related to the production of hydroxyl radicals through Fenton processes. However, the presence of magnetite slightly slows down the decarboxylation kinetics of formic acid in an aqueous solution of hydrogen peroxide. This behavior has been attributed to the possible formation of Fe-bearing hydroxyl formate aqueous species that could serve as stable transition states, leading to a decrease in the activation entropy of formic acid decomposition.

From these results, we can infer that the degradation processes of organic matter caused by oxidants are rather complex and understanding the underlying mechanisms is far from straightforward. In the case of Mars, it is also fundamental to figure out the interplay among oxidants, minerals, and ionizing radiations to comprehensively address the problem of preservation of organic compounds. In the next section, we will review some of the most relevant studies regarding the stability of organic compounds under Martian-like conditions.

### 4.2. Photodegradation Processes of Organic Matter on Mars

#### 4.2.1. Radiation Environment on Mars

Several studies have been performed so far to investigate the radiation environment on Mars, which has critical implications in the emergence and evolution of possible life forms on the planet.

The current very thin (~6 mbar) atmosphere of Mars, dominated by carbon dioxide (CO_2_), is capable of absorbing photons of wavelengths shorter than 190 nm [47,159], with the absorption cross section for carbon dioxide of the order of 10^−23^ cm^2^ at 195 nm, 10^−18^ cm^2^ in the range 130–150 nm, and 10^−17^ cm^2^ in the range 98–120 nm [159,160], and also does not allow efficient penetration of X-rays [11,161,162]. Thus, only mid- and near UV may cause degradation of organic compounds at the surface, and the respective fluxes have been both modelled and measured *in situ* (for more detail, see references [46,47,48,49,50,163]).

Gamma-rays and other high-energy heavy particles from cosmic rays, instead, are capable of penetrating the Martian atmosphere and the subsurface [164,165]. Dartnell et al. [165] calculated the dose-depth profile from galactic cosmic rays in the Martian subsurface considering a variety of factors, like variations of surface composition (dry regolith, ice, layered permafrost), solar minimum and maximum conditions, locations of different elevation (Olympus Mons, Hellas basin, datum altitude), and increasing atmospheric thickness over geological history. Matthiä et al. provided the most recent overview of model calculations about the highly energetic primary cosmic radiation at the Martian surface and comparison to *in situ* measurements by the MSL-Radiation Assessment Detector (MSL-RAD) on board the Curiosity rover [166]. Specifically, model estimates for the dose rate reach from 171 μGy/d to 307 μGy/d compared to a measured value of 233 ± 12 μGy/d [166].

**Figure 3:**
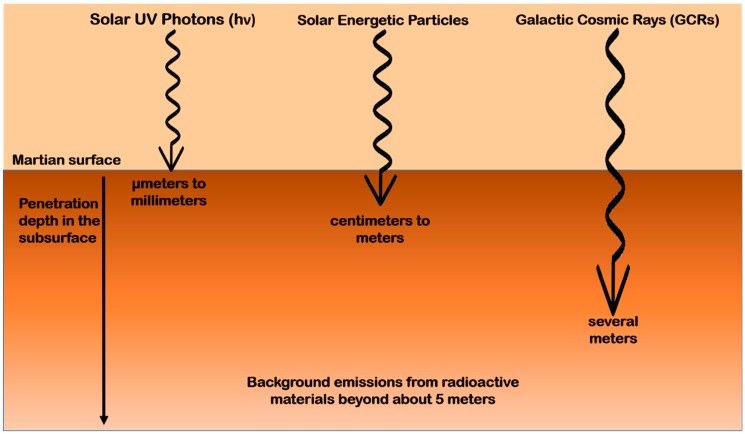
Penetration depths of different kinds of radiation in the near-surface of Mars.

The effects of such ionizing radiations depend on the penetration depth in the Martian subsurface, estimated as up to two meters for X-rays and energetic solar particles *vs.* a few microns or millimeters for UV radiation [67,167,168,169,170,171,172]. Instead, under a layer of snow or of H_2_O/CO_2_ ice, it seems that UV radiation can also penetrate up to meters according to radiative transfer models and laboratory experiments [173,174]. As depicted in Figure 3, molecules within a micrometer to a millimeter of the Martian surface may likely be altered by UV solar photons [175,176]. From few millimeters to a few centimeters, or even a meter, degradation may be caused by solar energetic particles [170]. From at least a meter to several meters, galactic cosmic rays and their secondary electrons may be responsible for degradation. At higher depths, like 4–5 m, radionuclides in the ground provide the most significant source of ionizing radiations [164].

#### 4.2.2. Laboratory Simulations of Martian Conditions

The stability of organic molecules under high-irradiation environments has been widely investigated (see ten Kate, 2010 [177], and references therein). However, studies not taking into account the effects of mineral phases cannot be considered a realistic simulation of the Martian conditions, since the interactions of organic molecules with minerals may completely change their reaction pathways [57]. Minerals can mediate or enhance the effects of ionizing radiations, and it is extremely important to figure out the roles of minerals in the variety of photoprocesses that may occur on Mars to investigate the preservation of possible biomarkers.

Table 2 summarizes some of the most relevant studies carried out so far about the stability of organic molecules in the presence of minerals simulating Martian conditions.

##### Early Investigations

In 1979, Oró and Holzer reported one of the first studies about the effects of Martian UV irradiation on biomarkers [178]. In this study, adenine, glycine, and naphthalene were adsorbed on powdered quartz (SiO_2_) and irradiated with mid-UV light both in a dry nitrogen (N_2_) atmosphere and at various oxygen (O_2_) concentrations and exposure times, along with the Murchison meteorite. Under an N_2_ atmosphere, adenine and glycine were very photostable during the entire duration of the irradiation experiment, while naphthalene and the volatilizable and pyrozable content of the Murchison meteorite showed degradation. In the presence of O_2_, instead, a significant increase in the degradation rate was observed for all samples, suggesting that oxidizing species may have an important role in the degradation of organic compounds. Subsequently, Stoker and Bullock measured the rate of photodecomposition of glycine mixed with a palagonitic regolith under a simulated Martian atmosphere and observed that this significantly exceeds the rate of organic deposition on Mars by meteoritic infall [176]. Specifically, they estimated a quantum efficiency for the decomposition of glycine in the spectral range of 200-240 nm of 1.46 ± 1.0 × 10^−6^ molecules/photon, corresponding to a destruction rate on Mars of 2.24 ± 1.2 × 10^−4^ g m^−2^ per year [176].

Ten Kate et al. investigated the stability of thin films of glycine and D-alanine subjected to far and mid-UV light under vacuum (4 × 10^−6^ mbar) at room temperature, finding half-lifetimes of 22 ± 5 h and 3 ± 1 h, respectively, and predicted that the half-lifetimes of the amino acids are extended to the order of 10^7^ years when embedded into regolith with a mixing ratio of 1 ppb [175]. The latter is in agreement with the results of Oró and Holzer for glycine decomposition in the presence of low levels of oxygen [178], suggesting that radical-induced decomposition mechanisms may have also played a role in the experiments performed by ten Kate et al. due to, for example, the incorporation of a limited amount of water in the amino acid thin films during deposition and/or irradiation that may generate reactive radicals upon irradiation.

However, it is worth noting that each one of these laboratory studies lack some of the factors that allow proper simulation of the Martian environment. For example, experiments at room temperature may overestimate destruction rates with respect to low temperatures currently existing on Mars, while different results may be obtained at different atmospheric conditions or in the absence of minerals simulating the Martian regolith.

In a subsequent investigation, ten Kate et al. inspected separately the effects of a CO_2_ atmosphere and low temperature on the destruction rate of glycine irradiated with mid-UV light, but without minerals [191]. The results show that the presence of a 7 mbar CO_2_ atmosphere does not affect the destruction rate of glycine compared to vacuum conditions, while a temperature of −63 °C (representing the average Martian temperature) slows down the degradation by a factor of 7 due to slower reaction kinetics. Scaling to Martian UV flux at noontime, they estimated a half-lifetime of 250 h for cold thin films of glycine, which is about one order of magnitude longer than previously evaluated [175] due to a temperature decrease of approximately 90 °C. This value of half-lifetime is also in agreement with subsequent studies performed by Stalport et al. [192] and Poch et al. [193]. ten Kate et al. explained the negligible effect of the 7 mbar CO_2_ atmosphere in their experiments based on the observation that the UV flux of their lamp was much higher than the rates of formation of oxygen radicals and UV extinction through scattering and absorption by CO_2_ over the full spectral range of their lamp [191]. The effect of temperature was completely attributed to slower degradation kinetics at lower temperatures, ruling out any involvement of the water accreted onto the glycine film at −63 °C, since that amount of water was too small to justify a significant UV absorption or production of hydroxyl radicals in the mid-UV spectral range in the absence of minerals [191].

##### Degradation Power of Water

A study by Garry et al. highlighted that water condensed onto minerals at low temperature may enhance degradation of amino acids [181]. Specifically, they investigated the stability of native amino acids of two analogs of Martian soil, JSC Mars-1 and Salten Skov. In experiment I and II, samples were exposed for 24 h and seven days, respectively, to mid-UV irradiation in vacuum at room temperature. In experiment III, new samples of the same materials were irradiated for seven days at low temperature (−63 °C) in a 7 mbar CO_2_ atmosphere. Experiments I and II revealed a slight increase in the concentration of amino acids, such as L-aspartic acid, L-leucine, L-glutamic acid, and L-alanine, probably due to the degradation of microorganism contaminations in the soils. At the low temperatures of experiment III, instead, the destruction of amino acids was observed, supporting the idea that the condensation of water onto the soil may be responsible for the production of reactive radical species, which in turn may be involved in the degradation of organics.

The catalytic activity of the Mars analog soil, JSC Mars-1, was pointed out also during the UVolution experiment flown on board the Biopan ESA module in 2007, in which the carboxylic acids, α-aminoisobutyric acid (AIB), mellitic acid, phthalic acid, and trimesic acid, were exposed to space conditions (UV radiation >200 nm) in low Earth orbit for 12 days both directly and underneath a layer of the Martian soil analog, JSC Mars-1 [185]. In the absence of JSC Mars-1, the half-lifetimes extrapolated to the Martian UV flux were in the range of 44–1062, 218–925, 122–317, and 19–169 h, for AIB, phthalic, trimesic, and mellitic acids, respectively. In the presence of JSC Mars-1, the photodestruction rate increased, giving half-lifetimes of 643 ± 317 and 506 ± 64 h for AIB and phthalic acid, respectively. No data are available for mixtures of trimesic acid and martian soil analogue, as the authors failed to prepare suitable samples in due time before the mission. Mellitic acid showed instability in the presence of the soil, producing a non-radiotolerant compound. Stalport et al. explained these results based on the catalytic activity of the titanium dioxide contained in the JSC Mars-1 soil [185]. Titanium dioxide (TiO_2_) may be involved in a variety of photochemical processes, such as the production of reactive oxygen species, like superoxides and hydroxyl radicals, capable of degrading organic compounds [152], and the photocatalytic synthesis of prebiotic organic molecules, including carboxylic acids, amino acids, nucleobases, and intermediates of the citric acid cycle [66,194,195]. A recent study about rutile, the most common and stable form of TiO_2_ that is ubiquitous on Earth and Mars, demonstrated that natural rutile with impurities and oxygen vacancies has a narrowed band gap and several intermediate levels in the forbidden band [196]. Therefore, it is also able to create excitons under exposure to visible light, which explains the high photocatalytic activity of natural rutile under solar irradiation. The conduction band electrons and valence band holes may enable processes, like the photo-reduction of CO_2_ to organic molecules (e.g., acetic acid and CH_4_) and the photo-oxidative generation of oxidants (e.g., OH•, O_2_ and ClO_4_^−^) via rutile photocatalysis [196].

Johnson and Pratt investigated the effects of UV radiation and ions of magnesium, calcium, sodium, and iron, with respect to the rates of diagenesis of amino acids, like glycine, L-alanine, L-valine, L-glutamic acid, and L-aspartic acid, in metal-rich sulfate brines analogous to the Martian ones [186]. This study was supposed to mimic saline systems that could have existed during warmer and wetter periods of Mars’ history or later periods of diagenesis, when saline groundwater percolated through the Martian bedrock. Results revealed that, when exposed to metal-rich brine solutions, the amino acids would undergo rapid oxidation and racemization on time scales of a few years. Such investigation allowed analysis of the both oxidation and racemization mechanisms. Regarding oxidation, they estimated very similar oxidation rates for all amino acids, despite the differential stability of the free radical alpha amino acids that are intermediate in the oxidation process [197]. Indeed, one would expect a lower destruction rate for glycine based on its less-stable primary radical intermediate with respect to secondary or tertiary alpha amino acids. These results were ascribed to a greater binding efficiency of glycine to metals in solution [198] and/or preferential incorporation within precipitates [199]. Similarly, Johnson and Pratt argued that the rates of oxidation of alanine and valine should be lower relative to aspartic and glutamic acids due to the poor electron-withdrawing nature of their aliphatic side-chain constituents, as observed in the stabilization of carbanion intermediates during racemization [200]. However, they found oxidation rates for alanine, valine, and aspartic acid within the range of 1 × 10^−3^ and 4 × 10^−3^ h^−1^ in all sample brines studied except for glutamic acid, in which oxidation doubles those values in the presence of iron. The greater oxidation of glutamic acid was not interpreted as the effect of photo-Fenton processes, which should actually favor oxidation of smaller, more aliphatic compounds, like alanine and valine [201,202], but other phenomena were considered, like differences in chelation due to the carboxylic acid side-chain constituent and correlation with the molecular weight. At the solution pH, glutamic acid has the possibility to form chelates both as a mono- and a bi-dentate species through the α-COO- and γ-COOH groups [203], thus possibly increasing electron transfer with the metal centers. This is true also for aspartic acid, but previous research has shown a positive correlation between molecular weight and degradation after exposure to ionizing radiation [170], which would explain the lower oxidation rate of aspartic acid with respect to glutamic acid. Lower molecular weight amino acids showed slightly increased oxidation with iron in solution, but only when exposed to UV radiation, which is indicative of a synergistic photo-Fenton oxidation mechanism. Regarding racemization, one would expect a trend similar to oxidation, since it occurs via the abstraction of the alpha carbon proton, forming a planar carbanion structure that can be re-protonated in either enantiomeric configuration [204]. However, overall, all amino acids showed a similar range of racemization rates, indicative of a similar metal-catalyzed reaction scheme. The racemization rates, on average, were estimated as an order of magnitude lower than oxidation rates, indicating a greater susceptibility of amino acids to radiolytic oxidation relative to racemization. Moreover, experimental outcomes revealed that the extent of racemization is independent both from the presence of iron and UV radiation. In the case of aspartic and glutamic acid, the observed increase in racemization in iron brines was attributed to increased complexation with metals.

The general trend emerging from these experiments is that the interaction with metals in solution can considerably increase both oxidation and racemization rates, likely due to iron and non-iron metals forming Fenton-type reaction pathways in such highly mineralized solutions [200].

These results imply that high water activity on Mars might have caused rapid oxidation of biologically relevant molecules within very short geological timescales, contrary to the classical “follow the water” principle, which Mars exploration missions have been based on. Nevertheless, preserved organic compounds might be found on Mars if they precipitated rapidly from source brines, experiencing minimal interaction with water, and were sequestered within “protective” mineral matrices before complete oxidation.

Similarly, Johnson and Pratt attested the “degradation power” of water also in another study of mid-UV irradiation under Martian-like atmosphere of L-alanine, L-valine, L-aspartic acid, L-glutamic acid, and glycine inoculated into a Martian regolith simulant, namely the Indiana-Mars Analog Regolith (I-MAR), mimicking high-silica andesitic basalts [187]. Analysis of amino acid recovery from the I-MAR samples pointed to a limited effect of UV radiation below the uppermost few millimeters of regolith. Using the model by Garry et al. for irradiation of a column of regolith [181], they estimated a penetration depth of 56 μm. The decrease in amino acid concentration at depths below the UV penetration depth was ascribed to processes different from photolytic oxidation. Specifically, at depths below 2 cm, they observed a delay in the amino acid concentration loss, both in the irradiated and dark samples, which implies a diffusion-controlled mechanism for the oxidation of these compounds, migrating downward through the regolith during the course of the experiment. Interestingly, the rate of loss of these compounds appeared to be very similar to the ones for photolytic or radiolytic oxidation (10^−7^–10^−8^ s^−1^) [170,175], suggesting a similar reaction mechanism, like the formation of hydrogen peroxide and hydroxyl radicals, by the diffusion and accumulation of atmospheric water vapor condensed at mineral grain boundaries during low temperature cycles in the experiments. Hurowitz et al. observed that the reaction of basaltic surfaces with minor amounts of water can produce measurable quantities of hydrogen peroxide in the absence of UV radiation or gas phases [150]. Johnson and Pratt calculated that in their experiments, the regolith surface might have equilibrated approximately with a uniform, single layer of water molecules [187], similarly to the predictions of a few monolayers of water adsorbed permanently to the Martian regolith [205]. They concluded that amino acid oxidation would thus be dependent on the rate of water vapor diffusion into the regolith and the availability of reactive basaltic surfaces.

##### Photocatalysis

Photocatalytic activity has been denoted also in the case of the hydrated metal oxides, anatase, goethite, and hematite, toward degradation of carboxylic, hydroxycarboxylic, and aminocarboxylic acids, carboxylated aromatics, amino acids, and peptides in aqueous suspensions [183].

In particular, Shkrob and Chemerisov carried out irradiation experiments both at room (22 °C) and low temperatures (from −196 to −73 °C) using a UV wavelength of 355 nm, which is absorbed only by the oxide mineral particles. At low temperatures, a more efficient photocatalytic decomposition of the carboxylated molecules was observed due to more favorable binding to mineral surface sites. Data indicated that the main photodegradation path is decarboxylation initiated by charge transfer from the metal oxide to the adsorbate, and anatase (TiO_2_), goethite (FeO(OH)), and hematite (Fe_2_O_3_) feature a similar photocatalytic activity for aromatic, carboxylic, and hydroxycarboxylic acids, while for α-amino acids and peptides, hematite has reduced activity. Further investigations indicated the formation of carbon dioxide and methane during the process of photocatalytic decarboxylation via the photo-Kolbe reaction [184], which is easily initiated through absorption of UVA (315–400 nm) by oxide minerals and takes place at low temperatures. This kind of process likely occurs on Mars at the surface of particulate iron(III) oxides abundant in the Martian regolith, and provides a viable route for seasonally variable methane production on Mars.

Shkrob et al. also inspected the photocatalytic oxidation of aqueous suspensions of nucleic acid components with anatase (TiO_2_), goethite (FeO(OH)), and hematite (Fe_2_O_3_) [188]. Purine radical cations and sugar-phosphate radicals formed as a consequence of the oxidation of purine nucleotides, while in the case of pyrimidine nucleotides, other than thymine, only the sugar-phosphate moiety underwent oxidation. The oxidation of the thymine derivatives resulted in deprotonation from the methyl group of the base. A difference was denoted between some single-stranded (ss) oligoribonucleotides and wild-type ss RNA that were oxidized at purine sites, and double-stranded (ds) oligoribonucleotides and DNA that conversely showed high stability against oxidation. These observations imply that duplex DNA would be better preserved on Mars since it is more resistant to oxidative diagenesis. From a prebiotic point of view, ds DNA might have been naturally selected as the “molecule of life” for its polymer morphology, and has radical chemistry favorable to withstand oxidative stress with respect to ss RNA, provided that metal oxides served as a template for synthesis of polynucleotides.

##### Clay Minerals

Photoprotective properties, instead, have been shown in several studies involving clay minerals.

Scappini et al. investigated the effect of UV radiation at 266 nm on aqueous solutions of free and clay-adsorbed DNA [179]. It turned out that the clay minerals, montmorillonite ((Na,Ca)_0.3_(Al,Mg)_2_Si_4_O_10_(OH)_2_·n(H_2_O)) and kaolinite (Al_2_Si_2_O_5_(OH)_4_), are able to protect DNA, reducing the radiation damage with respect to free DNA. They argued that such a photoprotective effect should not be associated to a mechanical shielding because the adsorption of the nucleic acids takes place only on the surface of the clay mineral [206]. Morphological and chemical factors should be involved, instead, like a change in DNA configuration from B to A when adsorbed on the mineral surface. The B configuration is much more compact and its binding to the surface sites may take place through electrostatic and/or hydrogen bonds, likely stabilizing the molecule. With a similar experiment, Biondi et al. demonstrated the ability of montmorillonite to protect the catalytic RNA molecule, ADHR1 (Adenine Dependent Hairpin Ribozyme 1), from UV-induced damages [182].

Ciaravella et al. observed a similar photoprotective effect also, exposing aqueous suspensions of DNA, montmorillonite, and kaolinite to soft X-rays (1.49, 4.51 and 8.04 keV) for exposure times ranging from 2 min up to 16 h [180]. Specifically, they proved that free DNA is damaged by X-rays at a level depending on the energy dose rather than the hardness of the radiation. On the contrary, in the presence of clay minerals, DNA is not damaged by X-rays for energy doses up to 5.8 × 10^4^ erg.

Poch et al. carried out mid-UV irradiation experiments under Martian-like temperatures and pressures on glycine and adenine molecules co-deposited with the iron(III)-smectite clay, nontronite ((CaO_0.5_,Na)_0.3_Fe^3+^_2_(Si,Al)_4_O_10_(OH)_2_·nH_2_O), from aqueous solution, at very high molecule-mineral mass ratio (from 1.0 to 3.6), to simulate the evaporation of small, warm ponds of liquid water containing a high concentration of organics [172]. Such a high concentration of organics is not ideal when interested in studying the catalytic/protective properties of minerals, since only a part of the total organic molecules can establish direct physico-chemical interactions with the mineral surface sites while the other molecules form multilayers on the surface, which make it more difficult to discern between multiple possible effects. In fact, in this situation, photolysis of the molecules directly exposed to the UV radiation can occur simultaneously to photoprotection mechanisms provided by the mineral or by the molecules adsorbed in the upper layers, as well as transformation processes of the molecules as a consequence of their interaction with the mineral, which can act as either a photocatalyst or a stoichiometric reagent. The results of their study revealed a reduction of the efficiencies of photodecomposition of glycine and adenine by a factor of 5 in the presence of nontronite, along with additional photoprotection by a factor of 5, doubling the amount of nontronite in the sample of glycine. These observations strongly suggest that the photoprotection provided by nontronite is not only due to mechanical shielding, but also a sort of stabilizing molecule-mineral interaction taking place, such as electrostatic interactions of the molecules in the interlayers and/ or on the edges of nontronite, allowing a more efficient energy dissipation and/or easier recombination for the fragments of the photo-dissociated molecules. Neither formation of photoproducts nor catalytic or stoichiometric degradation caused by surface groups of the mineral matrix were observed. However, nontronite promoted an acceleration of the dissociation of urea, perhaps due to its greater ability to chelate Fe^3+^ ions with respect to glycine and adenine, which may be responsible for a more efficient photo-oxidation and decomposition.

The catalytic behavior of clay minerals was evidenced also by Otroshchenko and Vasilyeva with another singular experiment, in which a dry powder of montmorillonite ((Na,Ca)_0.3_(Al,Mg)_2_Si_4_O_10_(OH)_2_·n(H_2_O)) was first irradiated with UV light at ambient conditions for 6 h, and then mixed with an acidic aqueous solution of adenosine monophosphate (AMP) [207]. The suspension was kept under stirring at 4–5 °C for 24 h, followed by centrifugation and extraction of the molecules from the clay. The analysis of the extract showed the formation of tetranucleotides. Therefore, Otroshchenko and Vasilyeva observed that adsorption of AMP on preliminarily UV-irradiated clay can initiate the formation of oligonucleotides that have linear chains with the typical 3′-5′ inter-nucleotide bonds of natural nucleic acids. The authors stated that similar results were obtained also upon the irradiation of SiO_2_ or volcanic ashes, and using guanosine monophosphate (GMP), but they did not show the relative data [207]. The suggested mechanism of formation of oligonucleotides involves the photolysis of the water molecules bound to the minerals in the presence of UV radiation. This results in the formation of hydrogen peroxide that, in turn, decomposes to give reactive radicals, eventually attacking the weakest 2′ and 3′ sites of the ribose cycle and forming the inter-nucleotide linkage. This hypothesis was verified by incubating for 24 h at 4 °C a non-irradiated sample of AMP adsorbed onto montmorillonite in a solution containing hydrogen peroxide, and observing the formation of more complex species [207]. However, the authors did not provide detailed data, so the mechanism remains unclear.

An important highlight of these studies is that a straightforward classification of Martian minerals as catalytic or protective ones is not possible, since the behavior of minerals under Martian conditions may depend on the specific organic molecules involved and their specific interactions with the mineral surface sites. This points out the need for a scrutinized systematic study of a variety of mineral matrices in combination with many organic compounds belonging to a wide range of molecular classes under simulated Martian conditions.

##### Towards Realistic Simulations of the Martian Environment

Dos Santos et al. studied under simulated Mars conditions, the preservation of 25 amino acids spiked onto several Mars-relevant minerals, like olivine ((Mg,Fe)_2_SiO_4_), enstatite (MgSiO_3_), goethite (FeO(OH)), hematite (Fe_2_O_3_), gypsum (CaSO_4_·2H_2_O), jarosite (KFe^3+^_3_(OH)_6_(SO_4_)_2_), labradorite ((Ca,Na)(Si,Al)_4_O_8_), augite ((Ca,Na)(Mg,Fe,Al,Ti)(Si,Al)_2_O_6_), the smectites montmorillonite ((Na,Ca)_0.3_(Al,Mg)_2_Si_4_O_10_(OH)_2_·n(H_2_O)), nontronite ((CaO_0.5_,Na)_0.3_Fe^3+^_2_(Si,Al)_4_O_10_(OH)_2_·nH_2_O), and saponite (Ca_0.25_(Mg,Fe)_3_((Si,Al)_4_O_10_)(OH)_2_·n(H_2_O)), as well as a basaltic lava [189]. Their results confirmed that clay minerals feature protective properties towards amino acids. This behavior was explained based on typical features of clay minerals, like their high surface area, which favors molecular adsorption; their small pore sizes that limits the penetration of radiations; and their optimal interlayer sites for accommodation of organic compounds, creating a shielded environment against external agents. The sulfates, gypsum and jarosite, also protected amino acids, likely due to their low UV absorbance or entrapment of amino acids upon recrystallization of partially dissolved sulfate. On the other hand, as previously reported, minerals containing iron(II), like augite, enstatite, hematite, and basaltic lava, demonstrated photocatalytic activity. The high amino acid preservation observed for olivine in comparison to augite, enstatite, and basaltic lava was ascribed to the low content of iron(II) as measured by XRD [189]. Their data also highlighted that degradation of both D- and L-amino acids takes place at the same rate, and there is a correlation between the preservation/degradation of amino acids in the presence of UV radiation and their molecular structure; specifically, the amino acids with alkyl substitution in the α-carbon feature a greater photostability. Moreover, they observed that amino acid preservation increases as the amino acid concentration increases. This trend may be due to stabilizing inter-molecular interactions, or the formation of molecular aggregates in which some molecules are directly exposed to radiation while others are covered and more protected, or occupation of less exposed mineral sites.

Other relevant studies on an important class of biomarkers, namely the nucleic acid components, have been recently performed by Ertem et al. [190] and Fornaro et al. [67].

Ertem et al. analyzed the photostability of purine, pyrimidine, and uracil under conditions mimicking Martian mid-UV irradiation, atmosphere, and the presence of oxidants, like sodium perchlorate (NaClO_4_) [190]. They pointed out some protection capability of the minerals, calcite (CaCO_3_), calcium sulfate (CaSO_4_), kaolinite (Al_2_Si_2_O_5_(OH)_4_), and clay-bearing Atacama desert soil. In the presence of these minerals, they observed molecular degradation of only 1–2% in contrast to complete degradation in the absence of the minerals when molecules were subjected directly to a UV flux equivalent to only five Martian day’s exposure. These organic compounds appeared to be very unstable also in the presence of ferric oxide, decomposing completely before UV irradiation into products without any chromophore group. Noteworthy, the presence of 0.6% sodium perchlorate did not cause any effect on the degradation outcomes. Similarly, results of the UV irradiation experiment carried out at 15–25 mbar and at ambient pressure were comparable and demonstrated that pressure has no significant effect on the irradiation products, in accordance with previous studies by Horneck et al. [208] and Schuerger et al. [209,210].

Fornaro et al. carried out a series of *in situ* and ex situ mid-UV irradiation experiments about the preservation of nucleic acid components in different conditions: (i) 25 °C/vacuum (~10^−2^–10^−3^ mbar); (ii) ambient terrestrial temperature and pressure; and (iii) −20 °C/6 mbar CO_2_ [67,168]. In particular, the nucleobases, adenine, uracil, cytosine, and hypoxanthine, were irradiated with mid-UV light *in situ* at 25 °C under vacuum, both as pure solid powder and adsorbed on the minerals, magnesium oxide (MgO) and forsterite (Mg_2_SiO_4_) [168]. Results showed that cytosine and hypoxanthine have a greater photostability compared to adenine and uracil because no evidence of significant degradation was observed under the experimental conditions, both in the case of pure compounds and nucleobases adsorbed onto magnesium oxide and forsterite. In the case of adenine and adenine adsorbed on the minerals, slight degradation was observed, while significant changes in the infrared spectra during UV irradiation were denoted for uracil both pure and adsorbed on the minerals. Comparison of the degradation kinetics of the same vibrational modes for the pure nucleobases and the nucleobases adsorbed on the minerals showed that minerals make degradation faster and more probable (the half-lifetimes of degradation decrease and the degradation cross sections increase), as acting as catalysts. In the case of uracil, new infrared features indicative of possible photoproducts appeared during UV irradiation. These new bands appeared at exactly the same wavenumbers both for pure uracil and uracil adsorbed on forsterite, but their formation was faster in the presence of the mineral. A revaluation of these data in light of new experiments [67] allowed the assignment of these features to a cis-syn cyclobutane dimer, whose formation occurs through a [2 + 2] cycloaddition of the C5C6 double bonds of adjacent pyrimidine bases. Forsterite is supposed to catalyze such a reaction by concentrating the molecules on a local scale through adsorption and inducing the correct orientation of reactive groups of neighboring molecules through specific molecule-mineral interactions. Thus, this kind of catalytic activity is likely related to a proximity effect. The same photoproduct was also observed upon *in situ* UV irradiation of uridine 5′-monophosphate (UMP) under terrestrial ambient conditions [67]. Consistently with studies reported in the literature, adenine and adenosine 5′-monophosphate (AMP) proved to be more stable in the presence of UV radiation thanks to the electronic structure of their chromophore, which determines an extremely efficient relaxation of the excited state [211] and proves more resistance to oxidation [212]. The photodynamics of the pyrimidine bases is much richer and the deactivation time from the excited state to the ground state is also much longer as compared to the purine bases, increasing the probability of photochemical reactions [213]. Interestingly, when irradiating AMP and UMP adsorbed on the serpentine mineral, lizardite (Mg_3_Si_2_O_5_(OH)_4_), under terrestrial ambient conditions, no new infrared features ascribable to cis-syn cyclobutane dimers were detected, but a new peak at 2164 cm^−1^ appeared, which was assigned to a cyanate molecular fragment, OCN-. Such a species has some relevance in the prebiotic context because cyanate may be involved in the synthesis of key biomolecules, like amino acids and polypeptides. It is worth noting that only in the presence of atmospheric oxygen there was evidence of its formation, perhaps due to the action of reactive oxygen radicals produced by UV-photolysis of O_2_. Different outcomes were obtained at −20 °C under a 6 mbar CO_2_ atmosphere; specifically, the degradation kinetics was on average three orders of magnitude slower than estimated under terrestrial ambient conditions, with half-lifetimes of the order of 50–100 Martian years for UMP and AMP both pure and adsorbed on lizardite, and no cyanate spectroscopic feature was observed. This suggests that current Martian conditions of temperatures below 0 °C and 6 mbar carbon dioxide atmosphere aid to stabilize important “building blocks of life”, such as nucleotides subjected to UV irradiation, likely due to the absence of reactive molecular oxygen and slower degradation kinetics. The temperature effect has been already verified by ten Kate et al. [191], who observed half-lifetimes for glycine about one order of magnitude longer than previously evaluated [175] due to a temperature decrease of roughly 90 °C. In the study of Fornaro et al. [67], one would expect even a less significant effect since the temperature variation was only 45 °C. Hence, the three orders of magnitude difference should be partly attributed also to the different atmospheric conditions. ten Kate et al. [191] argued that CO_2_ has no effect, with respect to vacuum, on the degradation kinetics, but this new study indicates that there is definitely a remarkable difference between an oxygenated and a non-oxygenated atmosphere. Consistent with previous experiments under terrestrial conditions and in vacuum, there were spectroscopic changes for pure UMP (not mixed with mineral) also under CO_2_ atmosphere at −20 °C, ascribable to the formation of cyclobutane dimers. Hence, the formation of cyclobutane dimers appears to be independent of the environmental conditions. This confirms the intrinsic higher photoreactivity of the uracil derivatives that might have been not selected in DNA, the “molecule of life”, due to their greater inclination to other reaction pathways. Fornaro et al. also compared the catalytic/protective properties of a variety of minerals relevant to Mars mineralogy [67]. They observed that labradorite ((Ca,Na)(Si,Al)_4_O_8_) and natrolite (Na_2_Al_2_Si_3_O_10_·2H_2_O) feature a remarkable catalytic activity, likely due to photo-ionization phenomena that may occur inside the mineral matrix, promoting redox processes. Hematite (Fe_2_O_3_) and forsterite (Mg_2_SiO_4_) showed an intermediate behavior, perhaps caused by the predominance of the effect related to the opacity of iron to UV radiation over the typical high reactivity of iron-bearing minerals. Apatite (Ca_5_(PO_4_)_3_(F,Cl,OH)), lizardite (Mg_3_Si_2_O_5_(OH)_4_), and antigorite ((Mg,Fe)_3_Si_2_O_5_(OH)_4_) did not show any significant catalytic effect. In the case of apatite, the photoprotection mechanism may be related to its capability to absorb UV radiation and efficiently dissipate energy via radiative decay. The serpentine minerals, lizardite and antigorites, are phyllosilicates characterized by high surface areas and optimal interlayer sites for adsorption and shielding of organic molecules. These findings are in agreement with the UV irradiation studies of amino acids adsorbed on various minerals under Martian-like conditions carried out by dos Santos et al. [189], revealing an overall greater preservation potential in the case of phyllosilicate minerals with respect to iron oxides (e.g., hematite) and feldspars (e.g., labradorite).

##### Effects of Galactic Cosmic Rays and Solar Energetic Particles

Galactic cosmic rays and solar energetic particles represent other important degrading agents for organic matter on Mars.

Pavlov et al. [214] and Dartnell et al. [164,165] have suggested that their influence extends much more in the subsurface than UV radiation, reaching a depth of several meters.

Clark [215] showed that cosmic rays may reach depths of 9 m on Mars based on its atmospheric column density that is only 16–27 g cm^−2^ at normal incidence (much lower than Earth’s atmospheric shield of 1000 g cm^−2^). However, despite their much higher penetration depth, laboratory experiments show that amino acids, like glycine, alanine, and phenylalanine, both in the presence and absence of water ice, would have a half-lifetime due to proton bombardment on the surface of Mars of about 10^8^ years (without taking into account additional effects, such as photolysis by UV photons) [169,216].

Similarly, Kminek and Bada showed that gamma radiations cause degradation of simple organic molecules, like the amino acids, L-aspartic acid, L-glutamic acid, glycine, L-alanine, and γ-amino-n-butyric acid, as well as methylamine and ethylamine, on timescales of hundreds of millions of years, and below a radiation shielding depth of 400–500 g cm^−2^, amino acids would not be substantially degraded due to the relatively low radiation dose from radioactive decay [170].

Conversely, other experiments of gamma irradiation of amino acids adsorbed on clay showed a low yield of recovery for tryptophan, aspartic acid, and glutamic acid by using the same source of gamma rays, and radiation doses two orders of magnitude smaller, but doubling the dose rate [217]. Montmorillonite ((Na,Ca)_0.3_(Al,Mg)_2_Si_4_O_10_(OH)_2_·n(H_2_O)) demonstrated some protection behavior against molecular decomposition with respect to the case of the pure amino acids in aqueous solutions, but not at a high extent. Greater photoprotection was observed in the case of adenine, which resisted high-radiation doses without transformation inside the clay [218]. In aqueous solution, the decomposition of the target compounds and the synthesis of other molecules by deamination/hydroxylation reactions may be promoted by the products derived from water radiolysis.

Another study about the effects of gamma radiation on mixtures of purine and uracil with calcium carbonate (CaCO_3_) reported a 10–13% loss of organics upon exposure to 3 Gy gamma rays that correspond to approximately 22 Martian years [190].

The presence of oxidants in the Martian soil also may have a great impact on the fate of organics irradiated with gamma rays or energetic solar particles.

Quinn et al. observed that calcium perchlorate exposed to gamma rays decomposes in a CO_2_ atmosphere to form hypochlorite (ClO^−^), chlorine dioxide (ClO_2_), and trapped oxygen (O_2_), which is an oxidizing species [133]. Gobi et al. explored the radiolytic decomposition of glycine under simulated Martian conditions in the presence of perchlorates by energetic electrons at 10, 160, 210, and 260 K, mimicking secondary electrons originating from the interaction of galactic cosmic rays with the Martian regolith in the first 5–10 cm depths over about 250 million years [137]. The experimental outcomes revealed that the presence of perchlorate has a significant effect on the decomposition rates of glycine, which increase by a factor of about two with respect to pure glycine. This indicates that, in the presence of perchlorates, two degradation mechanisms take place simultaneously: Radiolysis by the electrons and oxidation by the oxygen atoms released from the perchlorate.

Gobi et al. also highlighted that the degradation rates are independent of temperature (at least within the range of temperatures relevant for Mars, i.e., 160–260 K) [137]. Furthermore, they observed that the degradation rates of glycine are significantly higher than the formation rates of CO_2_ and CO, suggesting the occurrence of additional degradation pathways, such as a polymerization of glycine. In a subsequent study, Goby et al. unraveled the degradation mechanism for glycine, observing that decarboxylation is exclusively the first decay step during irradiation regardless of the presence of perchlorate anions [219]. In addition, they detected in pure glycine samples the decarboxylation co-product, methylamine (CH_3_NH_2_), and its radiolytic decay product, ammonia (NH_3_). In the presence of perchlorates, partial oxidation of methylamine may occur, which makes the decarboxylation equilibrium reaction of glycine irreversible. Thus, the depletion of the decarboxylation co-product, methylamine, results in an overall 10-fold increase in the formation rate of CO_2_ and its elevated concentrations in the perchlorate-containing irradiated samples.

Goby et al. further explored the effects of irradiation with energetic electrons on the stability of adenine mixed with magnesium perchlorate (Mg(ClO_4_)_2_) [136]. Also, in this case, the results indicated an increase of the destruction rate of adenine in the presence of perchlorate. This is likely due to the opening of alternative reaction channels, including the concurrent radiolysis/oxidation of the sample, resulting in a lot of radiolysis products, like carbon dioxide (CO_2_), isocyanic acid (HNCO), isocyanate (OCN^−^), carbon monoxide (CO), and nitrogen monoxide (NO), an oxidation product containing carbonyl groups (R_1_R_2_–C = O) with a constrained five-membered cyclic structure, and cyanamide (H_2_N–C ≡ N).

## 5. Summary of the Results and Take-Home Messages

Understanding the catalytic/protective behavior of Martian minerals is fundamental in order to unravel the possible prebiotic processes that might have led to the origin of life on Mars. Several experiments have been conducted to simulate the harsh Martian environment, especially by using UV sources or oxidants. This contribution has provided a review of the most relevant studies about the roles of minerals in the preservation of biomarkers under Martian-like conditions.

Table 3 specifically summarizes the irradiation experiments carried out so far on varied molecule-mineral complexes, along with the supposed mechanism of protection/catalysis, in the mid-UV spectral range that is the most relevant for Mars, and produces effects on very short timescales.

From these investigations, it turns out that establishing the conditions for preservation of organics on Mars is very challenging because the behavior of a mineral as a catalyst or protector strongly depends on the nature of the biomarker, the characteristics of the mineral itself, and the experimental conditions (e.g., molecule-mineral ratio, presence of oxidants, atmosphere, pressure, temperature, source of radiation, and so on). In general, the electronic structure of the molecules adsorbed on a mineral surface changes, influencing as a consequence the possible reaction pathways [220]. Photocatalytic minerals accelerate degradation of adsorbed molecules or catalyze radiation-driven reactions towards more complex species [57]. For instance, molecular photolysis may be favored by adsorption on the mineral surfaces because molecule-mineral interactions can weaken intramolecular bonds. The catalytic activity of some minerals may be related to a proximity effect, i.e., their ability to increase the local concentration of molecules through adsorption onto the mineral surfaces and, consequentially, the probability of molecular self-association and chemical reactions [57]. Moreover, molecular adsorption may take place with a configuration that facilitates interactions between reactive functional groups of molecules close to each other on the surface. Other minerals possess catalytic sites, such as transition metals, which are extremely active in electron transfer thanks to their *d* orbitals being only partially filled, thus may be easily involved in redox reactions. Indeed, such minerals undergo electron-hole separation upon absorption of photons, which can be followed by electron transfer to adsorbed molecules [220]. Furthermore, Fenton and photo-Fenton processes may occur with iron-bearing minerals. On the other hand, some minerals may provide preservation thanks to their structural properties, as observed in the case of phyllosilicates that feature optimal interlayer sites for molecular adsorption and mechanical shielding of organic compounds. Other minerals feature photoprotective properties based on peculiar electronic and optical features, like opacity to radiation, luminescence, low refractive index, etc., which reduce the damaging effects of ionizing radiations on adsorbed molecules [67,172,179,182,221].

Beyond UV irradiation, which produces significant effects at the surface, it has been shown that other ionizing radiations and energetic solar particles cause effects deeper in the subsurface of Mars, where a complex oxidation chemistry can take place. Therefore, in the actual Martian environment, various mechanisms play simultaneously and the overall stability of biomarkers under Martian conditions results from a subtle balance among them.

One of the important take-home messages is that it is extremely difficult to come up with a general mechanism to predict the behavior of the most relevant Martian minerals. Nevertheless, accurate investigations considering all the key factors for a realistic simulation of the Martian environment would provide an essential contribution to understand the trends.

In particular, from this review, it appears clear that laboratory simulations need to consider the presence of mineral phases and oxidizing agents representative of Martian regolith, the right source of ionizing radiations to reproduce the Martian irradiation environment, and the right temperatures, pressures, and atmospheric compositions, which influence the reaction kinetics, the chemical stability, and the physical state of the products [67,191,222].

Regarding ionizing radiations, the studies reported in the literature evidence the need to inspect the effects of UV radiation, since the degradation of organic compounds induced by UV light occurs much more rapidly, in days or months, with respect to higher-energy radiations and energetic solar particles, which take hundreds of millions of years, even though the penetration depth into the Martian regolith estimated for X-rays and energetic solar particles is much higher than UV, as previously mentioned [67,167,168,169,170,171,172]. The use of Xenon arc discharge lamps as a UV irradiation source has proven to better reproduce the energy and relative abundance of the UV photons in the spectral range of 190-400 nm that is most relevant for Mars, compared to mercury, hydrogen, or deuterium lamps that poorly match the solar irradiance at these wavelengths [168,209].

Regarding the possible effects of temperature, at the typical low temperatures on Mars, the condensation of water vapor on the regolith can supposedly give contrasting effects: Inhibition of the degradation of possible organic compounds thanks to UV absorption by the ice layer, or its enhancement thanks to the formation of radical species by water photodecomposition. It is worth noting that photodestruction of water into hydroxyl radicals does not occur at wavelengths above 190 nm [223], which are relevant for Mars’ surface photochemistry. Efficient photodecomposition of water is likely to occur only in the upper atmospheric layers of Mars, while production of reactive radical species due to mid-UV irradiation of water vapor close to the surface should not be significant. Nevertheless, it is not possible to exclude potential decomposition processes of water adsorbed on the Martian regolith, since the interaction of mid-UV with specific photocatalytic minerals may open new reaction pathways, leading to the formation of reactive radicals, like the hydroxyl ones. Hydroxyl radicals, in turn, can dimerize to give a strong oxidant, like hydrogen peroxide, which may be stable at significant depths within Martian regolith [146], and efficiently oxidize organic matter even at low temperatures thanks to its low thermal stability. Möhlmann [153,154] theoretically predicted the ubiquitous presence of a few monolayers of water on exposed Martian surfaces. Yen et al. [55] observed that superoxide radicals are formed through UV irradiation of feldspars under Martian-like conditions (−30 °C), in the presence of free oxygen and low concentrations of water. The quantum efficiency for this process has been estimated as 10^−6^ radicals/photon, corresponding to a production rate of 10^7^ cm^−2^ s^−1^. Moreover, in the case of iron-bearing minerals, as already mentioned, water adsorbed at the surface can leach soluble species, like Fe^2+^ ions, that may react with hydrogen peroxide in the presence of UV radiation through a photo-Fenton process, generating hydroxyl radicals. Therefore, organic degradation mechanisms driven by hydroxyl radicals produced by photolysis of water adsorbed on minerals can be considered plausible on Mars.

The presence of a CO_2_ atmosphere also may influence the stability of organics on Mars. CO_2_ efficiently absorbs far UV (below 190 nm), while for mid-UV, it presents a discrete absorption in the range of 190–203 nm; between 203 nm and 220 nm both scattering and absorption take place, and above 200 nm scattering becomes the dominant process [224,225]. The absorption of UV light above 167 nm may cause the dissociation of CO_2_ in carbon monoxide (CO) and oxygen radicals (O•), with a significant quantum yield only in the spectral range of 190–200 nm [223]. Given the low absorption of CO_2_ in this UV range, the upper limit for the formation rate of oxygen radicals is only 10^9^ s^−1^, which may result in a small effect on the overall molecular photostability. Conversely, it has been shown that CO_2_ can act as a scavenger of free radicals in specific conditions [226].

It would also be highly desirable to perform *in situ* analysis to avoid any alteration due to changes in environmental conditions, e.g., heating upon return to room temperature and pressure or contact with atmospheric oxygen and water vapor.

Moreover, since every analytical technique has its limitations and specificity, the use of only one analytical technique does not allow an in-depth understanding of the ongoing complicated mechanisms. Therefore, it would be worth developing new experimental apparatuses to perform *in situ* investigations with multiple analytical techniques.

## 6. Implications for Future Martian Missions

Laboratory simulations of Martian conditions are essential to make predictions about the mineral deposits with the highest preservation potential on Mars and, hence, select the most interesting sampling sites for future life detection missions on Mars.

Based on observations of terrestrial analog sites, the preferable locations to search for traces of life are sediments, evaporates, and hydrothermal systems because they are able to concentrate and better preserve biosignatures [113].

The long-term preservation of terrestrial biosignatures in several paleo-environments on Earth depends on the persistence of sedimentary materials; specifically, phosphates and silica, followed by clay-rich fine-grained sediments, carbonates, and metallic oxides, feature the highest preservation potential [116].

All candidate landing sites for the upcoming NASA Mars 2020 mission—i.e., Columbia Hills, Jezero crater, Northeast (NE) Syrtis, and NE Syrtis-Midway−show the presence of sedimentary rocks or possibly hydrothermal deposits, which may be favorable for the preservation of biomarkers [92,227,228,229]. Jezero and NE Syrtis present a high mineralogical diversity, including Mg-carbonates, pyroxenes, olivines, sulfates, Al-phyllosilicates, and clays, such as Fe/Mg smectites. Columbia Hills contains olivine materials, possible evaporates, ferric and calcium sulfates, Al-phyllosilicates, Mg/Fe carbonates, and opaline silica. Based on their mineralogy and lithology, all candidate landing sites for the NASA Mars 2020 mission can be considered plausible locations for the occurrence of prebiotic processes and possible emergence of complex biochemistry.

For the ESA ExoMars mission, the two candidate landing sites are [230]: (1) Oxia Planum, which features rather immature layers of vermiculite/di-tri octahedral Fe/Mg clays; and (2) Mawrth Vallis, which is characterized by a mature (almost lateritic) soil, a complex mixture comprising montmorillonite, beidellite, nontronite smectite, poorly crystalline aluminosilicate phases (allophane), and hydrated silica. Recent work has shown that organics may be particularly well stored in the topmost layer of the kind of soil present at Mawrth Vallis, within mixtures of oxides and allophane with Al-rich clays [109].

This study will also significantly benefit the 2020 Emirates Mars Mission (EMM) [231], China’s 2020 Mars mission [232], India’s 2022 Mars Orbiter Mission 2 (also called Mangalyaan 2) [233], and the JAXA Martian Moons Explorer (MMX) mission in 2024 [234]. Noteworthy, the MMX mission will collect samples from Phobos to return to Earth, which will be fundamental for determining the genesis of the Martian moons (specifically, whether they were once a part of Mars), and potentially contributing as another source of Martian biosignature information.

## Figures and Tables

**Table 1:** Relevant minerals detected on Mars, along with the region of discovery on Mars and the corresponding mission.

Minerals	Region of Discovery on Mars	Mars Mission
Phyllosilicates, such as clay minerals (aluminium-rich dioctahedral and magnesium-rich trioctahedral smectites, kaolinite, illite, pyrophyllite-talc), micas (muscovite), and chamosite chlorite.	Echus Chasma, Mawrth Vallis, Eridania basin, Memnonia quadrangle, Elysium quadrangle, Nili Fossae, Argyre Planitia, Gale crater, and Marathon Valley.	ESA Mars Express, NASA Mars Exploration Rovers, NASA Mars Reconnaissance Orbiter, and NASA Mars Science Laboratory [1,4,71,72,73,74,75,76,77,78,79,80].
Evaporites (chloride salts).	Southern highlands within Noachian-aged terrains.	NASA 2001 Mars Odyssey [87,88,89].
Sulfates (magnesium sulfates, like kieserite, and calcium sulfates, like gypsum).	Gale crater, Endeavor crater, the North and South Poles of Mars.	ESA Mars Express and NASA Mars Science Laboratory [69,90,91].
Opaline silica.	Valles Marineris, Gusev crater, and Gale crater.	NASA Mars Reconnaissance Orbiter and NASA Mars Science Laboratory [67,89,90].
Ferric oxides, like hematite and goethite.	Chryse Planitia, Utopia Planitia, Ares Vallis, Gusev Crater, Meridiani Planum, Terra Meridiani, Valles Marineris, Aureum Chaos, Columbia Hills, Gale crater.	NASA Viking Landers, NASA Mars Global Surveyor, NASA Mars Pathfinder, NASA 2001 Mars Odyssey, and NASA Mars Exploration Rovers [66,92,93,101].
Mafic minerals, such as pyroxene and olivine.	Mainly in older terrains, included within sand dunes, associated to ancient Noachian crustal rocks and early Hesperian volcanism in the southern hemisphere.	ESA Mars Express [105,106].
Amorphous material.	Gale crater.	NASA Mars Science Laboratory [86,108].

**Table 2:** Summary of the most relevant studies about the stability of organic molecules in the presence of minerals under Martian-like conditions.

Reference	Sample/Preparation Method	Irradiation Source/Spectral Range	Temperature	Pressure/Atmospheric Composition	Oxidants	*In Situ*/*Ex Situ* Analysis	Analytical Techniques
Oro & Holzer 1979 [178]	Adenine, glycine, and naphthalene impregnated on powdered quartz at various concentration from 0.01% to 0.2%/Murchison meteorite	Mercury-Xenon lamp/200–300 nm	−10 to 25 °C	1 mbar N_2_/various O_2_-content	None	*Ex situ*	Ion exchange chromatography for glycine, UV-vis spectrophotometry for adenine, Gas chromatography for naphthalene and Murchison
Stoker & Bullock 1997 [177]	Glycine powder mixed with palagonite at 1% concentration	Xenon lamp/210–710 nm	Room temperature	100 mbar, 95.59% CO_2_, 4.21% Ar, 0.11% O_2_, 0.09% CO	None	*In situ*	Gas chromatography
Scappini et al. 2004 [179]	Aqueous suspension of DNA and montmorillonite and kaolinite (20 μg DNA and 2 mg clay in 2 mL water)	Nd:YAG pulsed laser/266 nm	Room temperature	Terrestrial ambient conditions	None	*Ex situ*	Biological transformation
Ciaravella et al. 2004 [180]	Aqueous suspension of DNA and montmorillonite and kaolinite (10 μg DNA and 2 mg clay in 1.4 mL water)	Electron impact X-ray source/Monochromatic X-rays of 1.49, 4.51, and 8.04 keV	Room temperature	Vacuum	None		Biological transformation
Garry et al. 2006 [181]	JSC Mars-1 and Salten Skov Martian soil analogs containing native amino acids	Deuterium lamp/190–325 nm	Room temperature (Experiment I and II)/−63 °C (Experiment III)	1 × 10^−5^ mbar (Experiment I and II)/7 mbar CO_2_ (Experiment III)	None	*Ex situ*	HPLC
Biondi et al. 2007 [182]	Aqueous suspension of RNA and montmorillonite (2.25 × 10^−10^ moles RNA and 1.3 mg montmorillonite in 75 μL water)	Atlas Germicidal Lamp (15 W)/254 nm	Room temperature	Terrestrial ambient conditions	None	*Ex situ*	Analysis of self-cleavage activity
Shkrob & Chemerisov 2009 [183]	Aqueous suspensions of carboxylic, hydroxycarboxylic, and aminocarboxylic acids, carboxylated aromatics, amino acids, and peptides with anatase, goethite, and hematite	Nd:YAG pulsed laser/355 nm	−196 to −73 °C/22 °C	1 bar, N_2_	None	*Ex situ*	EPR/transient absorption spectroscopy
Shkrob et al. 2010 [184]	Aqueous suspensions of carboxylic, hydroxycarboxylic, and aminocarboxylic acids, carboxylated aromatics, amino acids, and peptides with anatase, goethite, and hematite	Nd:YAG pulsed laser/355 nm	−196 °C	1 bar, N_2_	None	*Ex situ*	EPR
Stalport et al. 2010 [185]	Carboxylic acids α-aminoisobutyric acid (AIB), mellitic acid, phthalic acid, and trimesic acid directly deposited on quartz windows or underneath a layer of JSC Mars-1	Solar radiation >200 nm	Temperature at low Earth orbit	Pressure at low Earth orbit	None	*Ex situ*	IR spectroscopy
Johnson & Pratt 2010 [186]	Amino acids glycine, L-alanine, L-valine, L-glutamic acid, and L-aspartic acid in metal-rich sulfate brines (1 mM amino acid concentration)	Xenon lamp/250–700 nm	−40 to 20 °C	7 to 15 mbar, 95.3% CO_2_, 2.7% N_2_, 1.6% Ar, and 0.13% O_2_	None	*Ex situ*	XRD, HPLC
Johnson & Pratt 2011 [187]	Amino acids L-Alanine, L-valine, L-aspartic acid, L-glutamic acid, and glycine inoculated into I-MAR Martian regolith simulant at 0.01% concentration	Xenon lamp/210–900 nm	−40.4 to 24 °C (on average −17.6 °C)	10^−22^ mbar (on average 13.3 mbar), 48.6% CO_2_, 50% Ar, 1.4% N_2_, 0.07% O_2_, 0.04% CO, 0.02% H_2_O, 0.01% H_2_	None	*Ex situ*	HPLC
Shkrob et al. 2011 [188]	Aqueous suspensions of nucleic acid components with anatase, goethite, and hematite	Nd:YAG pulsed laser/355 nm	−196 °C	1 bar, N_2_	None	*Ex situ*	EPR
Fornaro et al. 2013 [168]	Nucleobases adenine, uracil, cytosine, and hypoxanthine adsorbed on magnesium oxide and forsterite at concentrations in the range 0.1–10%	Mercury-Xenon lamp/185–2000 nm	25 °C	Vacuum (~10^−2^–10^−3^ mbar)	None	*In situ*	Diffuse Reflectance Fourier Transform Infrared (DRIFT) spectroscopy
Poch et al. 2015 [172]	Glycine, urea, and adenine co-deposited with nontronite with high molecule-mineral mass ratio (from 1.0 to 3.6)	Xenon lamp/190–400 nm	−55 °C	6 ± 1 mbar, N_2_	None	*In situ*	IR spectroscopy
dos Santos et al. 2016 [189]	25 amino acids spiked onto augite, enstatite, goethite, gypsum, hematite, jarosite, labradorite, montmorillonite, nontronite, olivine, saponite, and a basaltic lava, at various concentration (approx. 0.001% to 0.1%)	Xenon lamp/200–400 nm	−80 to 20 °C	6 mbar, 95% CO_2_, 5% N_2_	None	*Ex situ*	GC-MS
Ertem et al. 2017 [190]	Purine, pyrimidine, and uracil impregnated on ferric oxide, calcite, calcium sulphate, kaolinite, and clay-bearing Atacama desert soil at 0.0025% concentration	Xenon lamp/200–400 nm (Experiment I); Gamma Cell 40 from a ^137^Cs source/Gamma rays 3 Gy (Experiment II)	−196 to 25 °C (Experiment I); 25 °C (Experiment II)	15–25 mbar/Ambient pressure, 95.3% CO_2_, 2.7% N_2_, 0.13 O_2_ (Experiment I); Ambient pressure, 95.3% CO_2_, 2.7% N_2_, 0.13 O_2_ (Experiment II)	0.6% NaClO_4_	*Ex situ*	HPLC
Fornaro et al. 2018 [67]	AMP and UMP adsorbed on lizardite, antigorite, labradorite, natrolite, hematite, apatite, and forsterite at 5% concentration	Xenon lamp/200–930 nm (Experiment I); Xenon lamp/180–900 nm (Experiment II)	25 °C (Experiment I); −20 °C (Experiment II)	Terrestrial ambient conditions (Experiment I); 6 mbar CO_2_ (Experiment II)	None	*In situ* (Experiment I); *Ex situ* (Experiment II)	Diffuse Reflectance Fourier Transform Infrared (DRIFT) spectroscopy

**Table 3:** Supposed mechanisms of preservation/degradation of biomarkers due to the interactions with minerals under mid-UV irradiation.

Mineral	Biomarker	Radiation	Protection/Preservation	Catalysis/Degradation	Supposed Mechanism
Quartz	Adenine	200–300 nm	Under N_2_	Under O_2_	Photo-oxidation by O_2_ (Oro & Holzer 1979).
Quartz	Glycine	200–300 nm	Under N_2_	Under O_2_	Photo-oxidation by O_2_ (Oro & Holzer 1979).
Quartz	Naphthalene	200–300 nm	x	Under O_2_/N_2_	Photo-oxidation by O_2_ (Oro & Holzer 1979).
Murchison meteorite	Indigenous organics	200–300 nm	x	Under O_2_/N_2_	Photo-oxidation by O_2_ (Oro & Holzer 1979).
Palagonite	Glycine	210–710 nm	x	Under Martian-like atmosphere	Photolysis into CH_4_, C_2_H_6_, C_2_H_4_ (Stoker & Bullock 1997).
JSC Mars-1 and Salten Skov Martian soil analogs	Indigenous amino acids	190–325 nm	x	Under Martian-like atmosphere	Decomposition induced by radicals produced by photolysis of water condensed onto minerals (Garry et al. 2006).
JSC Mars-1 Martian soil analog	Carboxylic acids α-aminoisobutyric acid (AIB), mellitic acid, phthalic acid, and trimesic acid	Solar radiation > 200 nm	x	Under low Earth orbit conditions	Decomposition induced by radicals/oxidants produced by TiO_2_–photocatalysis (Stalport et al. 2010).
I-MAR Martian regolith simulant	Amino acids L-Alanine, L-valine, L-aspartic acid, L-glutamic acid, and glycine	210–900 nm	x	Under Martian-like atmosphere	Photolytic oxidation up to UV penetration depth, then decomposition induced by radicals formed from condensed atmospheric water vapor diffused into the regolith (Johnson & Pratt 2011).
Aqueous suspensions of anatase, goethite, and hematite	Carboxylic, hydroxycarboxylic, and aminocarboxylic acids, carboxylated aromatics, amino acids and peptides	355 nm	x	Under N_2_	Decarboxylation initiated by charge transfer from the metal oxide to the adsorbate. Specifically, anatase, goethite, and hematite feature a similar photocatalytic activity for aromatic, carboxylic, and hydroxycarboxylic acids, while for α-amino acids and peptides hematite has reduced activity (Shkrob et al. 2010).
Aqueous suspensions of anatase, goethite, and hematite	Nucleic acid components	355 nm	Only for double-stranded oligoribonucleotides and DNA	Under N_2_	Oxidation of purine nucleotides leads to formation of purine radical cations and sugar-phosphate radicals. In the case of pyrimidine nucleotides other than thymine only the sugar-phosphate moiety undergoes oxidation, while deprotonation from the methyl group of the base occurs for thymine derivatives. Single-stranded (ss) oligoribonucleotides and wild-type ss RNA are oxidized at purine sites, while double-stranded (ds) oligoribonucleotides and DNA show high stability against oxidation (Shkrob et al. 2011).
Aqueous suspensions of montmorillonite and kaolinite	DNA	266 nm	Under terrestrial ambient conditions	x	Photoprotection due to specific molecule-mineral interactions; specifically, a change in DNA configuration from B to A when adsorbed on the mineral surface, which is more compact and its binding to the surface sites may take place through electrostatic and/or hydrogen bonds likely stabilizing the molecule (Scappini et al. 2004).
Aqueous suspensions of montmorillonite	RNA molecule ADHR1	254 nm	Under terrestrial ambient conditions	x	Photoprotection due to specific molecule-mineral interactions (Biondi et al. 2007).
Nontronite	Glycine and adenine	190–400 nm	Under N_2_	x	Photoprotection is not only due to mechanical shielding, but also stabilizing molecule-mineral interactions, such as electrostatic interactions of the molecules in the interlayers and/ or on the edges of nontronite allowing a more efficient energy dissipation and/or easier recombination for the fragments of the photo-dissociated molecules (Poch et al. 2015).
Nontronite	Urea	190–400 nm	x	Under N_2_	Catalysis in urea photo-oxidation and decomposition, maybe due to chelation with Fe^3+^ ions (Poch et al. 2015).
Smectites montmorillonite, nontronite and saponite	25 Amino acids	200–400 nm	Under Martian-like conditions	x	Photoprotection by mechanical shielding effect (dos Santos et al. 2016).
Sulfates gypsum and jarosite	25 Amino acids	200–400 nm	Under Martian-like conditions	x	Photoprotection due to low UV absorbance of sulfates or entrapment of amino acids upon recrystallization of partially dissolved sulfate (dos Santos et al. 2016).
Augite, enstatite, hematite and basaltic lava	25 Amino acids	200–400 nm	x	Under Martian-like conditions	Photocatalytic activity due to iron(II) reactions (dos Santos et al. 2016).
Calcite, calcium sulphate, kaolinite, clay-bearing Atacama desert soil + 0.6% NaClO_4_	Purine, pyrimidine and uracil	200–400 nm	Under Martian-like conditions	x	Photoprotection mechanism not specified (Ertem et al. 2017).
Ferric oxide + 0.6% NaClO_4_	Purine, pyrimidine and uracil	200–400 nm	x	Under Martian-like conditions	Complete decomposition before UV irradiation (Ertem et al. 2017).
Magnesium oxide and forsterite	Adenine, uracil, cytosine, and hypoxanthine	185–2000 nm	x	Under vacuum	Catalysis likely due to a proximity effect (Fornaro et al. 2013).
Lizardite, antigorite and apatite	AMP and UMP	200–930 nm	Under Martian-like conditions	x	Various photoprotection mechanisms: mechanical shielding/stabilizing molecule-mineral interactions for lizardite and antigorite, photo-luminescence for apatite (Fornaro et al. 2018).
Labradorite, natrolite, hematite, forsterite	AMP and UMP	200–930 nm	x	Under Martian-like conditions	Remarkable catalytic activity of labradorite and natrolite, likely due to photo-ionization phenomena that may occur inside the mineral matrix promoting redox processes. For hematite and forsterite the catalytic activity is not so high, maybe due to the opacity of iron to UV radiation (Fornaro et al. 2018).

## References

[1] Grotzinger J.P., Sumner D.Y., Kah L.C., Stack K., Gupta S., Edgar L., Rubin D., Lewis K., Schieber J., Mangold N. (2014). A Habitable Fluvio-Lacustrine Environment at Yellowknife Bay, Gale Crater, Mars. Science.

[2] Arvidson R.E., Squyres S.W., Bell J.F., Catalano J.G., Clark B.C., Crumpler L.S., de Souza P.A., Fairén A.G., Farrand W.H., Fox V.K. (2014). Ancient Aqueous Environments at Endeavour Crater, Mars. Science.

[3] Sun V.Z., Milliken R.E. (2015). Ancient and Recent Clay Formation on Mars as Revealed from a Global Survey of Hydrous Minerals in Crater Central Peaks. J. Geophys. Res. Planets.

[4] Carter J., Poulet F., Bibring J.P., Mangold N., Murchie S. (2013). Hydrous Minerals on Mars as Seen by the CRISM and OMEGA Imaging Spectrometers: Updated Global View. J. Geophys. Res. Planets.

[5] Carter J., Poulet F., Bibring J.-P., Murchie S., Langevin Y., Mustard J.F., Gondet B. Phyllosilicates and Other Hydrated Minerals on Mars: Global Distribution as Seen by MEx/OMEGA. Proceedings of the 40th Lunar and Planetary Science Conference.

[6] Carter J., Loizeau D., Mangold N., Poulet F., Bibring J.-P. (2015). Widespread Surface Weathering on Early Mars: A Case for a Warmer and Wetter Climate. Icarus.

[7] L’Haridon J., Mangold N., Meslin P.-Y., Johnson J.R., Rapin W., Forni O., Cousin A., Payré V., Dehouck E., Nachon M. (2018). Chemical Variability in Mineralized Veins Observed by ChemCam on the Lower Slopes of Mount Sharp in Gale Crater, Mars. Icarus.

[8] Michalski J.R., Dobrea E.Z.N., Niles P.B., Cuadros J. (2017). Ancient Hydrothermal Seafloor Deposits in Eridania Basin on Mars. Nat. Commun..

[9] Feldman W.C., Prettyman T.H., Maurice S., Plaut J.J., Bish D.L., Vaniman D.T., Mellon M.T., Metzger A.E., Squyres S.W., Karunatillake S. (2004). Global Distribution of Near-Surface Hydrogen on Mars. J. Geophys. Res. Planets.

[10] Milliken R.E., Mustard J.F., Poulet F., Jouglet D., Bibring J.-P., Gondet B., Langevin Y. (2007). Hydration State of the Martian Surface as Seen by Mars Express OMEGA: 2. H_2_O Content of the Surface. J. Geophys. Res. Planets.

[11] Smith M.D., Wolff M.J., Lemmon M.T., Spanovich N., Banfield D., Budney C.J., Clancy R.T., Ghosh A., Landis G.A., Smith P. (2004). First Atmospheric Science Results from the Mars Exploration Rovers Mini-TES. Science.

[12] Orosei R., Lauro S.E., Pettinelli E., Cicchetti A., Coradini M., Cosciotti B., Di Paolo F., Flamini E., Mattei E., Pajola M. (2018). Radar Evidence of Subglacial Liquid Water on Mars. Science.

[13] Fisk M.R., Giovannoni S.J. (1999). Sources of Nutrients and Energy for a Deep Biosphere on Mars. J. Geophys. Res. Planets.

[14] McKay D.S., Gibson E.K., Thomas-Keprta K.L., Vali H., Romanek C.S., Clemett S.J., Chillier X.D., Maechling C.R., Zare R.N. (1996). Search for Past Life on Mars: Possible Relic Biogenic Activity in Martian Meteorite ALH84001. Science.

[15] Morris R.V., Ruff S.W., Gellert R., Ming D.W., Arvidson R.E., Clark B.C., Golden D.C., Siebach K., Klingelhöfer G., Schröder C. (2010). Identification of Carbonate-Rich Outcrops on Mars by the Spirit Rover. Science.

[16] Boynton W.V., Ming D.W., Kounaves S.P., Young S.M.M., Arvidson R.E., Hecht M.H., Hoffman J., Niles P.B., Hamara D.K., Quinn R.C. (2009). Evidence for Calcium Carbonate at the Mars Phoenix Landing Site. Science.

[17] Ehlmann B.L., Mustard J.F., Murchie S.L., Poulet F., Bishop J.L., Brown A.J., Calvin W.M., Clark R.N., Des Marais D.J., Milliken R.E. (2008). Orbital Identification of Carbonate-Bearing Rocks on Mars. Science.

[18] Eigenbrode J.L., Summons R.E., Steele A., Freissinet C., Millan M., Navarro-gonzález R., Sutter B., Mcadam A.C., Conrad P.G., Hurowitz J.A. (2018). Organic Matter Preserved in 3-Billion-Year-Old Mudstones at Gale Crater, Mars. Science.

[19] Stewart R.F., Jensen L.H. (1967). Redetermination of the Crystal Structure of Uracil. Acta Crystallogr..

[20] Stern J.C., Sutter B., Freissinet C., Navarro-González R., McKay C.P., Archer P.D., Buch A., Brunner A.E., Coll P., Eigenbrode J.L. (2015). Evidence for Indigenous Nitrogen in Sedimentary and Aeolian Deposits from the Curiosity Rover Investigations at Gale Crater, Mars. Proc. Natl. Acad. Sci. USA.

[21] Steele A., McCubbin F.M., Fries M.D. (2016). The Provenance, Formation, and Implications of Reduced Carbon Phases in Martian Meteorites. Meteorit. Planet. Sci..

[22] Fogel M.L., Steele A. (2013). Nitrogen in Extraterrestrial Environments: Clues to the Possible Presence of Life. Elements.

[23] McCubbin F.M., Jones R.H. (2015). Extraterrestrial Apatite: Planetary Geochemistry to Astrobiology. Elements.

[24] Powner M.W., Gerland B., Sutherland J.D. (2009). Synthesis of Activated Pyrimidine Ribonucleotides in Prebiotically Plausible Conditions. Nature.

[25] Szostak J.W. (2009). Origins of Life: Systems Chemistry on Early Earth. Nature.

[26] Freissinet C., Glavin D.P., Mahaffy P.R., Miller K.E., Eigenbrode J.L., Summons R.E., Brunner A.E., Buch A., Szopa C., Archer P.D. (2015). Organic Molecules in the Sheepbed Mudstone, Gale Crater, Mars. J. Geophys. Res. Planets.

[27] Farquhar J., Savarino J., Jackson T.L., Thiemens M.H. (2000). Evidence of Atmospheric Sulphur in the Martian Regolith from Sulphur Isotopes in Meteorites. Nature.

[28] Vago J.L., Westall F., Cavalazzi B., Team T.E.S.W., Cavalazzi B.W.F. (2019). Searching for Signs of Life on Other Planets: Mars a Case Study. Biosignatures for Astrobiology.

[29] Westall F., Bost N., Vago J.L., Kminek G., Campbell K.A. (2015). Biosignatures on Mars: What, Where, and How? Implications for the Search for Martian Life. Astrobiology.

[30] Steele A., McCubbin F.M., Fries M., Kater L., Boctor N.Z., Fogel M.L., Conrad P.G., Glamoclija M., Spencer M., Morrow A.L. (2012). A Reduced Organic Carbon Component in Martian Basalts. Science.

[31] Glavin D.P., Freissinet C., Miller K.E., Eigenbrode J.L., Brunner A.E., Buch A., Sutter B., Archer P.D., Atreya S.K., Brinckerhoff W.B. (2013). Evidence for Perchlorates and the Origin of Chlorinated Hydrocarbons Detected by SAM at the Rocknest Aeolian Deposit in Gale Crater. J. Geophys. Res. Planets.

[32] Flynn G.J. (1996). The Delivery of Organic Matter from Asteroids and Comets to the Early Surface of Mars. Earth Moon Planets.

[33] Frantseva K., Mueller M., ten Kate I.L., Van der Tak F.F.S., Greenstreet S. (2018). Delivery of Organics to Mars through Asteroid and Comet Impacts. Icarus.

[34] Flynn G.J., Nittler L.R., Engrand C. (2016). Composition of Cosmic Dust: Sources and Implications for the Early Solar System. Elements.

[35] Grady M.M., Verchovsky A.B., Wright I.P. (2004). Magmatic Carbon in Martian Meteorites: Attempts to Constrain the Carbon Cycle on Mars. Int. J. Astrobiol..

[36] Wright I.P., Grady M.M., Pillinger C.T. (1992). Chassigny and the Nakhlites: Carbon-Bearing Components and Their Relationship to Martian Environmental Conditions. Geochim. Cosmochim. Acta.

[37] Vago J., Witasse O., Svedhem H., Baglioni P., Haldemann A., Gianfiglio G., Blancquaert T., McCoy D., de Groot R. (2015). ESA ExoMars Program: The next Step in Exploring Mars. Sol. Syst. Res..

[38] Mahaffy P.R., Webster C.R., Cabane M., Conrad P.G., Coll P., Atreya S.K., Arvey R., Barciniak M., Benna M., Bleacher L. (2012). The Sample Analysis at Mars Investigation and Instrument Suite. Space Sci. Rev..

[39] Millan M., Szopa C., Buch A., Coll P., Glavin D.P., Freissinet C., Navarro-Gonzalez R., Francois P., Coscia D., Bonnet J.Y. (2016). In Situ Analysis of Martian Regolith with the SAM Experiment during the First Mars Year of the MSL Mission: Identification of Organic Molecules by Gas Chromatography from Laboratory Measurements. Planet. Space Sci..

[40] Miller K.E., Eigenbrode J.L., Freissinet C., Glavin D.P., Kotrc B., Francois P., Summons R.E. (2016). Potential Precursor Compounds for Chlorohydrocarbons Detected in Gale Crater, Mars, by the SAM Instrument Suite on the Curiosity Rover. J. Geophys. Res. Planets.

[41] Franz H.B., McAdam A.C., Ming D.W., Freissinet C., Mahaffy P.R., Eldridge D.L., Fischer W.W., Grotzinger J.P., House C.H., Hurowitz J.A. (2017). Large Sulfur Isotope Fractionations in Martian Sediments at Gale Crater. Nat. Geosci..

[42] Steele A., Benning L.G., Wirth R., Siljestrom S., Fries M.D., Hauri E., Conrad P.G., Rogers K., Eigenbrode J., Schreiber A. (2018). Organic Synthesis on Mars by Electrochemical Reduction of CO_2_. Sci. Adv..

[43] Mustard J., Adler M., Allwood A., Bass D., Beaty D., Bell J., Brinckerhoff W., Carr M., Des Marais D., Drake B. (2013). Report of the Mars 2020 Science Definition Team. Space Safety Magazine.

[44] Córdoba-Jabonero C., Lara L., Mancho A., Márquez A., Rodrigo R. (2003). Solar Ultraviolet Transfer in the Martian Atmosphere: Biological and Geological Implications. Planet. Space Sci..

[45] Martínez G.M., Newman C.N., De Vicente-Retortillo A., Fischer E., Renno N.O., Richardson M.I., Fairén A.G., Genzer M., Guzewich S.D., Haberle R.M. (2017). The Modern Near-Surface Martian Climate: A Review of In-Situ Meteorological Data from Viking to Curiosity. Space Sci. Rev..

[46] Patel M.R., Bérces A., Kolb C., Lammer H., Rettberg P., Zarnecki J.C., Selsis F. (2003). Seasonal and Diurnal Variations in Martian Surface Ultraviolet Irradiation: Biological and Chemical Implications for the Martian Regolith. Int. J. Astrobiol..

[47] Patel M.R., Zarnecki J.C., Catling D.C. (2002). Ultraviolet Radiation on the Surface of Mars and the Beagle 2 UV Sensor. Planet. Space Sci..

[48] Vicente-Retortillo Á., Lemmon M.T., Martínez G.M., Valero F., Vázquez L., Martín L. (2016). Seasonal and Interannual Variability of Solar Radiation at Spirit, Opportunity and Curiosity Landing Sites. Fís. Tierra.

[49] Vicente-Retortillo Á., Martínez G.M., Renno N.O., Lemmon M.T., de la Torre-Juárez M. (2017). Determination of Dust Aerosol Particle Size at Gale Crater Using REMS UVS and Mastcam Measurements. Geophys. Res. Lett..

[50] Vicente-Retortillo Á., Valero F., Vázquez L., Martínez G.M. (2015). A Model to Calculate Solar Radiation Fluxes on the Martian Surface. J. Space Weather Space Clim..

[51] Matthiä D., Ehresmann B., Lohf H., Köhler J., Zeitlin C., Appel J., Sato T., Slaba T., Martin C., Berger T. (2016). The Martian Surface Radiation Environment—A Comparison of Models and MSL/RAD Measurements. J. Space Weather Space Clim..

[52] Clark B.C., Kounaves S.P. (2016). Evidence for the Distribution of Perchlorates on Mars. Int. J. Astrobiol..

[53] Kounaves S.P., Chaniotakis N.A., Chevrier V.F., Carrier B.L., Folds K.E., Hansen V.M., McElhoney K.M., O’Neil G.D., Weber A.W. (2014). Identification of the Perchlorate Parent Salts at the Phoenix Mars Landing Site and Possible Implications. Icarus.

[54] Georgiou C.D., Sun H.J., McKay C.P., Grintzalis K., Papapostolou I., Zisimopoulos D., Panagiotidis K., Zhang G., Koutsopoulou E., Christidis G.E. (2015). Evidence for Photochemical Production of Reactive Oxygen Species in Desert Soils. Nat. Commun..

[55] Yen A.S., Kim S.S., Hecht M.H., Frant M.S., Murray B. (2000). Evidence That the Reactivity of the Martian Soil Is Due to Superoxide Ions. Science.

[56] Sutter B., McAdam A.C., Mahaffy P.R., Ming D.W., Edgett K.S., Rampe E.B., Eigenbrode J.L., Franz H.B., Freissinet C., Grotzinger J.P. (2017). Evolved Gas Analyses of Sedimentary Rocks and Eolian Sediment in Gale Crater, Mars: Results of the Curiosity Rover’s Sample Analysis at Mars Instrument from Yellowknife Bay to the Namib Dune. J. Geophys. Res. Planets.

[57] Brucato J.B., Fornaro T., Cavalazzi B., Westall F. (2018). Role of Mineral Surfaces in Prebiotic Processes and Space-like Conditions. Biosignatures for Astrobiology.

[58] Fornaro T., Brucato J.R., Branciamore S., Pucci A. (2013). Adsorption of Nucleic Acid Bases on Magnesium Oxide (MgO). Int. J. Astrobiol..

[59] Fornaro T., Brucato J.R., Feuillie C., Sverjensky D.A., Hazen R.M., Brunetto R., D’Amore M., Barone V. (2018). Binding of Nucleic Acid Components to the Serpentinite-Hosted Hydrothermal Mineral Brucite. Astrobiology.

[60] Lambert J.-F. (2015). Origins of Life: From the Mineral to the Biochemical World. BIO Web Conf..

[61] Hazen R.M., Sverjensky D.A. (2010). Mineral Surfaces, Geochemical Complexities, and the Origins of Life. Cold Spring Harb. Perspect. Biol..

[62] Hazen R.M. (2006). Mineral Surfaces and the Prebiotic Selection and Organization of Biomolecules. Am. Mineral..

[63] Wächtershäuser G. (1990). Evolution of the First Metabolic Cycles. Proc. Natl. Acad. Sci. USA.

[64] Hazen R.M. (2005). Genesis: The Scientific Quest for Life’s Origin.

[65] Brandes J.A., Boctor N.Z., Cody G.D., Cooper B.A., Hazen R.M., Yoder H.S. (1998). Abiotic Nitrogen Reduction on the Early Earth. Nature.

[66] Botta L., Bizzarri B.M., Piccinino D., Fornaro T., Brucato J.R., Saladino R. (2017). Prebiotic Synthesis of Carboxylic Acids, Amino Acids and Nucleic Acid Bases from Formamide under Photochemical Conditions. Eur. Phys. J. Plus.

[67] Fornaro T., Boosman A., Brucato J.R., ten Kate I.L., Siljeström S., Poggiali G., Steele A., Hazen R.M. (2018). UV Irradiation of Biomarkers Adsorbed on Minerals under Martian-like Conditions: Hints for Life Detection on Mars. Icarus.

[68] Ehlmann B.L., Edwards C.S. (2014). Mineralogy of the Martian Surface. Annu. Rev. Earth Planet. Sci..

[69] Bibring J.P., Langevin Y., Mustard J. (2006). Global Mineralogical and Aqueous Mars History Derived from OMEGA/Mars Express Data. Science.

[70] Milliken R.E., Swayze G.A., Arvidson R.E., Bishop J.L., Clark R.N., Ehlmann B.L., Green R.O., Grotzinger J.P., Morris R.V., Murchie S.L. (2008). Opaline Silica in Young Deposits on Mars. Geology.

[71] Mustard J.F., Murchie S.L., Pelkey S.M., Ehlmann B.L., Milliken R.E., Grant J.A., Bibring J., Poulet F., Bishop J., Dobrea E.N. (2008). Hydrated Silicate Minerals on Mars Observed by the Mars Reconnaissance Orbiter CRISM Instrument. Nature.

[72] Murchie S.L., Mustard J.F., Ehlmann B.L., Milliken R.E., Bishop J.L., McKeown N.K., Noe Dobrea E.Z., Seelos F.P., Buczkowski D.L., Wiseman S.M. (2009). A Synthesis of Martian Aqueous Mineralogy after 1 Mars Year of Observations from the Mars Reconnaissance Orbiter. J. Geophys. Res. Planets.

[73] Negron-Mendoza A., Ramos-Bernal S., Seckbach J. (2004). The Role of Clays in the Origin of Life. Origins. Cellular Origin, Life in Extreme Habitats and Astrobiology.

[74] Ehlmann B.L., Mustard J.F., Fassett C.I., Schon S.C., Head J.W., Des Marais D.J., Grant J.A., Murchie S.L. (2008). Clay Minerals in Delta Deposits and Organic Preservation Potential on Mars. Nat. Geosci..

[75] Ehlmann B.L., Mustard J.F., Swayze G.A., Clark R.N., Bishop J.L., Poulet F., Des Marais D.J., Roach L.H., Milliken R.E., Wray J.J. (2009). Identification of Hydrated Silicate Minerals on Mars Using MRO-CRISM: Geologic Context near Nili Fossae and Implications for Aqueous Alteration. J. Geophys. Res. Planets.

[76] Ehlmann B.L., Mustard J.F., Murchie S.L., Bibring J.-P., Meunier A., Fraeman A.A., Langevin Y. (2011). Subsurface Water and Clay Mineral Formation during the Early History of Mars. Nature.

[77] Ehlmann B.L., Mustard J.F., Clark R.N., Swayze G.A., Murchie S.L. (2011). Evidence for Low-Grade Metamorphism, Hydrothermal Alteration, and Diagenesis on Mars from Phyllosilicate Mineral Assemblages. Clays Clay Miner..

[78] Bishop J.L., Dobrea E.Z.N., McKeown N.K., Parente M., Ehlmann B.L., Michalski J.R., Milliken R.E., Poulet F., Swayze G.A., Mustard J.F. (2008). Phyllosilicate Diversity and Past Aqueous Activity Revealed at Mawrth Vallis, Mars. Science.

[79] Noe Dobrea E.Z., Wray J.J., Calef F.J., Parker T.J., Murchie S.L. (2012). Hydrated Minerals on Endeavour Crater’s Rim and Interior, and Surrounding Plains: New Insights from CRISM Data. Geophys. Res. Lett..

[80] Adeli S., Hauber E., Le Deit L., Jaumann R. (2015). Geologic Evolution of the Eastern Eridania Basin: Implications for Aqueous Processes in the Southern Highlands of Mars. J. Geophys. Res. Planets.

[81] Vaniman D.T., Bish D.L., Ming D.W., Bristow T.F., Morris R.V., Blake D.F., Chipera S.J., Morrison S.M., Treiman A.H., Rampe E.B. (2014). Mineralogy of a Mudstone at Yellowknife Bay, Gale Crater, Mars. Science.

[82] Michalski J.R., Cuadros J., Bishop J.L., Darby Dyar M., Dekov V., Fiore S. (2015). Constraints on the Crystal-Chemistry of Fe/Mg-Rich Smectitic Clays on Mars and Links to Global Alteration Trends. Earth Planet. Sci. Lett..

[83] Bristow T.F., Bish D.L., Vaniman D.T., Morris R.V., Blake D.F., Grotzinger J.P., Rampe E.B., Crisp J.A., Achilles C.N., Ming D.W. (2015). The Origin and Implications of Clay Minerals from Yellowknife Bay, Gale Crater, Mars. Am. Mineral..

[84] Hurowitz J.A., Grotzinger J.P., Fischer W.W., McLennan S.M., Milliken R.E., Stein N., Vasavada A.R., Blake D.F., Dehouck E., Eigenbrode J.L. (2017). Redox Stratification of an Ancient Lake in Gale Crater, Mars. Science.

[85] Rampe E.B., Ming D.W., Blake D.F., Bristow T.F., Chipera S.J., Grotzinger J.P., Morris R.V., Morrison S.M., Vaniman D.T., Yen A.S. (2017). Mineralogy of an Ancient Lacustrine Mudstone Succession from the Murray Formation, Gale Crater, Mars. Earth Planet. Sci. Lett..

[86] Bristow T.F., Rampe E.B., Achilles C.N., Blake D.F., Chipera S.J., Craig P., Crisp J.A., Des Marais D.J., Downs R.T., Gellert R. (2018). Clay Mineral Diversity and Abundance in Sedimentary Rocks of Gale Crater, Mars. Sci. Adv..

[87] Osterloo M.M., Hamilton V.E., Bandfield J.L., Glotch T.D., Baldridge A.M., Christensen P.R., Tornabene L.L., Anderson F.S. (2008). Chloride-Bearing Materials in the Southern Highlands of Mars. Science.

[88] Osterloo M.M., Anderson F.S., Hamilton V.E., Hynek B.M. (2010). Geologic Context of Proposed Chloride-Bearing Materials on Mars. J. Geophys. Res. Planets.

[89] Glotch T.D., Bandfield J.L., Wolff M.J., Arnold J.A., Che C. (2016). Constraints on the Composition and Particle Size of Chloride Salt-Bearing Deposits on Mars. J. Geophys. Res. Planets.

[90] Squyres S.W. (2004). In Situ Evidence for an Ancient Aqueous Environment at Meridiani Planum, Mars. Science.

[91] McAdam A.C., Franz H.B., Sutter B., Archer P.D., Freissinet C., Eigenbrode J.L., Ming D.W., Atreya S.K., Bish D.L., Blake D.F. (2014). Sulfur-bearing Phases Detected by Evolved Gas Analysis of the Rocknest Aeolian Deposit, Gale Crater, Mars. J. Geophys. Res. Planets.

[92] Ruff S.W., Farmer J.D. (2016). Silica Deposits on Mars with Features Resembling Hot Spring Biosignatures at El Tatio in Chile. Nat. Commun..

[93] Viviano-Beck C.E., Seelos F.P., Murchie S.L., Kahn E.G., Seelos K.D., Taylor H.W., Morgan M.F. (2014). Revised CRISM Spectral Parameters and Summary Products Based on the Currently Detected Mineral Diversity on Mars. J. Geophys. Res. Planets.

[94] Gooding J.L. (1978). Chemical Weathering on Mars Thermodynamic Stabilities of Primary Minerals (and Their Alteration Products) from Mafic Igneous Rocks. Icarus.

[95] Christensen P.R., Bandfield J.L., Clark R.N., Edgett K.S., Hamilton V.E., Hoefen T., Kieffer H.H., Kuzmin R.O., Lane M.D., Malin M.C. (2000). Detection of Crystalline Hematite Mineralization on Mars by the Thermal Emission Spectrometer: Evidence for near-Surface Water. J. Geophys. Res. Planets.

[96] Christensen P.R., Morris R.V., Lane M.D., Bandfield J.L., Malin M.C. (2001). Global Mapping of Martian Hematite Mineral Deposits: Remnants of Water-Driven Processes on Early Mars. J. Geophys. Res. Planets.

[97] Arvidson R.E., Poulet F., Morris R.V., Bibring J.-P., Bell J.F., Squyres S.W., Christensen P.R., Bellucci G., Gondet B., Ehlmann B.L. (2006). Nature and Origin of the Hematite-Bearing Plains of Terra Meridiani Based on Analyses of Orbital and Mars Exploration Rover Data Sets. J. Geophys. Res. Planets.

[98] Catling D.C., Moore J.M. (2003). The Nature of Coarse-Grained Crystalline Hematite and Its Implications for the Early Environment of Mars. Icarus.

[99] Klingelhöfer G., Morris R.V., Bernhardt B., Schröder C., Rodionov D.S., de Souza P.A., Yen A., Gellert R., Evlanov E.N., Zubkov B. (2004). Jarosite and Hematite at Meridiani Planum from Opportunity’s Mössbauer Spectrometer. Science.

[100] Souza-Egipsy V., Ormö J., Bowen B.B., Chan M.A., Komatsu G. (2006). Ultrastructural Study of Iron Oxide Precipitates: Implications for the Search for Biosignatures in the Meridiani Hematite Concretions, Mars. Astrobiology.

[101] Bishop J.L., Fröschl H., Mancinelli R.L. (1998). Alteration Processes in Volcanic Soils and Identification of Exobiologically Important Weathering Products on Mars Using Remote Sensing. J. Geophys. Res. Planets.

[102] Koch C.B., Mørup S., Madsen M.B., Vistisen L. (1995). Iron-Containing Weathering Products of Basalt in a Cold, Dry Climate. Chem. Geol..

[103] Schwertmann U. (1958). The Effect of Pedogenic Environments on Iron Oxide Minerals. Advances in Soil Science.

[104] Fraeman A.A., Arvidson R.E., Catalano J.G., Grotzinger J.P., Morris R.V., Murchie S.L., Stack K.M., Humm D.C., McGovern J.A., Seelos F.P. (2013). A Hematite-Bearing Layer in Gale Crater, Mars: Mapping and Implications for Past Aqueous Conditions. Geology.

[105] Ody A., Poulet F., Bibring J.P., Loizeau D., Carter J., Gondet B., Langevin Y. (2013). Global Investigation of Olivine on Mars: Insights into Crust and Mantle Compositions. J. Geophys. Res. Planets.

[106] Ody A., Poulet F., Langevin Y., Bibring J.P., Bellucci G., Altieri F., Gondet B., Vincendon M., Carter J., Manaud N. (2012). Global Maps of Anhydrous Minerals at the Surface of Mars from OMEGA/MEx. J. Geophys. Res. Planets.

[107] Bibring J.-P., Langevin Y., Gendrin A., Gondet B., Poulet F., Berthé M., Soufflot A., Arvidson R., Mangold N., Mustard J. (2005). Mars Surface Diversity as Revealed by the OMEGA/Mars Express Observations. Science.

[108] Dehouck E., McLennan S.M., Meslin P.-Y., Cousin A. (2014). Constraints on Abundance, Composition, and Nature of X-Ray Amorphous Components of Soils and Rocks at Gale Crater, Mars. J. Geophys. Res. Planets.

[109] Basile-Doelsch I., Balesdent J., Rose J. (2015). Are Interactions between Organic Compounds and Nanoscale Weathering Minerals the Key Drivers of Carbon Storage in Soils?. Environ. Sci. Technol..

[110] Grady M.M., Wright I.P., Pillinger C.T. (1997). A Carbon and Nitrogen Isotope Study of Zagami. J. Geophys. Res. Planets.

[111] Steele A., Fries M.D., Amundsen H.E.F., Mysen B.O., Fogel M.L., Schweizer M., Boctor N.Z. (2007). Comprehensive Imaging and Raman Spectroscopy of Carbonate Globules from Martian Meteorite ALH 84001 and a Terrestrial Analogue from Svalbard. Meteorit. Planet. Sci..

[112] Steele A., McCubbin F.M., Fries M.D., Golden D.C., Ming D.W., Benning L.G. (2012). Graphite in the Martian Meteorite Allan Hills 84001. Am. Mineral..

[113] (2007). Committee on an Astrobiology Strategy for the Exploration of Mars.

[114] Hedges J.I., Keil R.G. (1995). Sedimentary Organic Matter Preservation: An Assessment and Speculative Synthesis. Mar. Chem..

[115] Lalonde K., Mucci A., Ouellet A., Gélinas Y. (2012). Preservation of Organic Matter in Sediments Promoted by Iron. Nature.

[116] Farmer J.D., Des Marais D.J. (1999). Exploring for a Record of Ancient Martian Life. J. Geophys. Res. Planets.

[117] Biondi E., Howell L., Benner S.A. (2017). Opal Absorbs and Stabilizes RNA–A Hierarchy of Prebiotic Silica Minerals. Synlett.

[118] Grotzinger J.P., Crisp J., Vasavada A.R., Anderson R.C., Baker C.J., Barry R., Blake D.F., Conrad P., Edgett K.S., Ferdowski B. (2012). Mars Science Laboratory Mission and Science Investigation. Space Sci. Rev..

[119] Gaillard F., Michalski J., Berger G., McLennan S.M., Scaillet B. (2013). Geochemical Reservoirs and Timing of Sulfur Cycling on Mars. Space Sci. Rev..

[120] Sephton M.A. (2002). Organic Compounds in Carbonaceous Meteorites. Nat. Prod. Rep..

[121] Kotler J.M., Hinman N.W., Yan B., Stoner D.L., Scott J.R. (2008). Glycine Identification in Natural Jarosites Using Laser Desorption Fourier Transform Mass Spectrometry: Implications for the Search for Life on Mars. Astrobiology.

[122] Aubrey A., Cleaves H.J., Chalmers J.H., Skelley A.M., Mathies R.A., Grunthaner F.J., Ehrenfreund P., Bada J.L. (2006). Sulfate Minerals and Organic Compounds on Mars. Geology.

[123] Fernández-Remolar D.C., Chong-Díaz G., Ruíz-Bermejo M., Harir M., Schmitt-Kopplin P., Tziotis D., Gómez-Ortíz D., García-Villadangos M., Martín-Redondo M.P., Gómez F. (2013). Molecular Preservation in Halite- and Perchlorate-Rich Hypersaline Subsurface Deposits in the Salar Grande Basin (Atacama Desert, Chile): Implications for the Search for Molecular Biomarkers on Mars. J. Geophys. Res. Biogeosci..

[124] Macko S.A., Engel M.H., Parker P.L. (1993). Early Diagenesis of Organic Matter in Sediments. Organic Geochemistry.

[125] Lasne J., Noblet A., Szopa C., Navarro-González R., Cabane M., Poch O., Stalport F., François P., Atreya S.K., Coll P. (2016). Oxidants at the Surface of Mars: A Review in Light of Recent Exploration Results. Astrobiology.

[126] Chevrier V.F., Hanley J., Altheide T.S. (2009). Stability of Perchlorate Hydrates and Their Liquid Solutions at the Phoenix Landing Site, Mars. Geophys. Res. Lett..

[127] Hecht M.H., Kounaves S.P., Quinn R.C., West S.J., Young S.M.M., Ming D.W., Catling D.C., Clark B.C., Boynton W.V., Hoffman J. (2009). Detection of Perchlorate and the Soluble Chemistry of Martian Soil at the Phoenix Lander Site. Science.

[128] Kounaves S.P., Carrier B.L., O’Neil G.D., Stroble S.T., Claire M.W. (2014). Evidence of Martian Perchlorate, Chlorate, and Nitrate in Mars Meteorite EETA79001: Implications for Oxidants and Organics. Icarus.

[129] Smith M.L., Claire M.W., Catling D.C., Zahnle K.J. (2014). The Formation of Sulfate, Nitrate and Perchlorate Salts in the Martian Atmosphere. Icarus.

[130] Carrier B.L., Kounaves S.P. (2015). The Origins of Perchlorate in the Martian Soil. Geophys. Res. Lett..

[131] Wang A., Jacson A., Yan Y.C., Houghton J. (Per)Chlorate Formation Through Electrochemistry in Martian Atmosphere-Surface Interaction. Proceedings of the 49th Lunar and Planetary Science Conference.

[132] Kolb V.M., Hoover R.B., Levin G.V., Rozanov A.Y., Retherford K.D. (2009). Oxidation of Organic Materials with Perchlorates: Relevance to the Chemistry on the Martian Surface.

[133] Quinn R.C., Martucci H.F.H., Miller S.R., Bryson C.E., Grunthaner F.J., Grunthaner P.J. (2013). Perchlorate Radiolysis on Mars and the Origin of Martian Soil Reactivity. Astrobiology.

[134] Crandall P.B., Góbi S., Gillis-Davis J., Kaiser R.I. (2017). Can Perchlorates Be Transformed to Hydrogen Peroxide (H_2_O_2_) Products by Cosmic Rays on the Martian Surface?. J. Geophys. Res. Planets.

[135] Góbi S., Bergantini A., Kaiser R.I. (2016). In Situ Detection of Chlorine Dioxide (ClO_2_) in the Radiolysis of Perchlorates and Implications for the Stability of Organics on Mars. Astrophys. J..

[136] Góbi S., Bergantini A., Kaiser R.I. (2017). Degradation of Adenine on the Martian Surface in the Presence of Perchlorates and Ionizing Radiation: A Reflectron Time-of-Flight Mass Spectrometric Study. Astrophys. J..

[137] Góbi S., Abplanalp M.J., Kaiser R.I. (2016). Effect of Perchlorates on Electron Radiolysis of Glycine with Application to Mars. Astrophys. J..

[138] Turner A.M., Abplanalp M.J., Kaiser R.I. (2016). Mechanistic Studies on the Radiolytic Decomposition of Perchlorates on the Martian Surface. Astrophys. J..

[139] Sutter B., Quinn R.C., Archer P.D., Glavin D.P., Glotch T.D., Kounaves S.P., Osterloo M.M., Rampe E.B., Ming D.W. (2017). Measurements of Oxychlorine Species on Mars. Int. J. Astrobiol..

[140] Clancy R., Sandor B., Moriarty-Schieven G. (2004). A Measurement of the 362 GHz Absorption Line of Mars Atmospheric H_2_O_2_. Icarus.

[141] Encrenaz T., Greathouse T.K., Lefèvre F., Atreya S.K. (2012). Hydrogen Peroxide on Mars: Observations, Interpretation and Future Plans. Planet. Space Sci..

[142] Encrenaz T., Greathouse T.K., Lefèvre F., Montmessin F., Forget F., Fouchet T., Dewitt C., Richter M.J., Lacy J.H., Bézard B. (2015). Astrophysics Seasonal Variations of Hydrogen Peroxide and Water Vapor on Mars: Further Indications of Heterogeneous Chemistry. Astron. Astrophys..

[143] Hunten D.M. (1979). Possible Oxidant Sources in the Atmosphere and Surface of Mars. J. Mol. Evol..

[144] Atreya S.K., Wong A.-S., Renno N.O., Farrell W.M., Delory G.T., Sentman D.D., Cummer S.A., Marshall J.R., Rafkin S.C.R., Catling D.C. (2006). Oxidant Enhancement in Martian Dust Devils and Storms: Implications for Life and Habitability. Astrobiology.

[145] Delory G.T., Farrell W.M., Atreya S.K., Renno N.O., Wong A.-S., Cummer S.A., Sentman D.D., Marshall J.R., Rafkin S.C.R., Catling D.C. (2006). Oxidant Enhancement in Martian Dust Devils and Storms: Storm Electric Fields and Electron Dissociative Attachment. Astrobiology.

[146] Bullock M.A., Stoker C.R., McKay C.P., Zent A.P. (1994). A Coupled Soil-Atmosphere Model of H_2_O_2_ on Mars. Icarus.

[147] Hartman H., McKay C.P. (1995). Oxygenic Photosynthesis and the Oxidation State of Mars. Planet. Space Sci..

[148] Zent A.P. (1998). On the Thickness of the Oxidized Layer of the Martian Regolith. J. Geophys. Res. Planets.

[149] Huguenin R.L., Miller K.J., Harwood W.S. (1979). Frost-Weathering on Mars: Experimental Evidence for Peroxide Formation. J. Mol. Evol..

[150] Hurowitz J.A., Tosca N.J., McLennan S.M., Schoonen M.A.A. (2007). Production of Hydrogen Peroxide in Martian and Lunar Soils. Earth Planet. Sci. Lett..

[151] Davila A.F., Fairén A.G., Gago-Duport L., Stoker C., Amils R., Bonaccorsi R., Zavaleta J., Lim D., Schulze-Makuch D., McKay C.P. (2008). Subsurface Formation of Oxidants on Mars and Implications for the Preservation of Organic Biosignatures. Earth Planet. Sci. Lett..

[152] Zent A.P., Ichimura A.S., Quinn R.C., Harding H.K. (2008). The Formation and Stability of the Superoxide Radical (O_2_^−^) on Rock-Forming Minerals: Band Gaps, Hydroxylation State, and Implications for Mars Oxidant Chemistry. J. Geophys. Res. Planets.

[153] Möhlmann D.T. (2004). Water in the Upper Martian Surface at Mid- and Low-Latitudes: Presence, State, and Consequences. Icarus.

[154] Möhlmann D.T.F. (2008). The Influence of van Der Waals Forces on the State of Water in the Shallow Subsurface of Mars. Icarus.

[155] Benner S.A., Devine K.G., Matveeva L.N., Powell D.H. (2000). The Missing Organic Molecules on Mars. Proc. Natl. Acad. Sci. USA.

[156] Mancinelli R.L. (1989). Peroxides and the Survivability of Microorganisms on the Surface of Mars. Adv. Space Res..

[157] McDonald G.D., de Vanssay E., Buckley J.R. (1998). Oxidation of Organic Macromolecules by Hydrogen Peroxide: Implications for Stability of Biomarkers on Mars. Icarus.

[158] Foustoukos D.I., Stern J.C. (2012). Oxidation Pathways for Formic Acid under Low Temperature Hydrothermal Conditions: Implications for the Chemical and Isotopic Evolution of Organics on Mars. Geochim. Cosmochim. Acta.

[159] Kuhn W.R., Atreya S.K. (1979). Solar Radiation Incident on the Martian Surface. J. Mol. Evol..

[160] Huestis D.L., Bougher S.W., Fox J.L., Galand M., Johnson R.E., Moses J.I., Pickering J.C. (2008). Cross Sections and Reaction Rates for Comparative Planetary Aeronomy. Space Sci. Rev..

[161] Smith D.S., Scalo J. (2007). Solar X-Ray Flare Hazards on the Surface of Mars. Planet. Space Sci..

[162] Jain R., Awasthi A.K., Tripathi S.C., Bhatt N.J., Khan P.A. (2012). Influence of Solar Flare X-Rays on the Habitability on the Mars. Icarus.

[163] Cockell C.S., Catling D.C., Davis W.L., Snook K., Kepner R.L., Lee P., Mckay C.P. (2000). The Ultraviolet Environment of Mars: Biological Implications Past, Present, and Future. Icarus.

[164] Dartnell L.R., Desorgher L., Ward J.M., Coates A.J. (2007). Modelling the Surface and Subsurface Martian Radiation Environment: Implications for Astrobiology. Geophys. Res. Lett..

[165] Dartnell L.R., Desorgher L., Ward J.M., Coates A.J. (2007). Martian Sub-Surface Ionising Radiation: Biosignatures and Geology. Biogeosci. Discuss..

[166] Matthiä D., Hassler D.M., de Wet W., Ehresmann B., Firan A., Flores-McLaughlin J., Guo J., Heilbronn L.H., Lee K., Ratliff H. (2017). The Radiation Environment on the Surface of Mars—Summary of Model Calculations and Comparison to RAD Data. Life Sci. Space Res..

[167] Schuerger A.C., Golden D.C., Ming D.W. (2012). Biotoxicity of Mars Soils: 1. Dry Deposition of Analog Soils on Microbial Colonies and Survival under Martian Conditions. Planet. Space Sci..

[168] Fornaro T., Brucato J.R., Pace E., Cestelli-Guidi M., Branciamore S., Pucci A. (2013). Infrared Spectral Investigations of UV Irradiated Nucleobases Adsorbed on Mineral Surfaces. Icarus.

[169] Gerakines P.A., Hudson R.L. (2013). Glycine’s Radiolytic Destruction in Ices: First in Situ Laboratory Measurements for Mars. Astrobiology.

[170] Kminek G., Bada J.L. (2006). The Effect of Ionizing Radiation on the Preservation of Amino Acids on Mars. Earth Planet. Sci. Lett..

[171] Poch O., Kaci S., Stalport F., Szopa C., Coll P. (2014). Laboratory Insights into the Chemical and Kinetic Evolution of Several Organic Molecules under Simulated Mars Surface UV Radiation Conditions. Icarus.

[172] Poch O., Jaber M., Stalport F., Nowak S., Georgelin T., Lambert J.-F., Szopa C., Coll P. (2015). Effect of Nontronite Smectite Clay on the Chemical Evolution of Several Organic Molecules under Simulated Martian Surface Ultraviolet Radiation Conditions. Astrobiology.

[173] Córdoba-Jabonero C., Zorzano M.-P., Selsis F., Patel M.R., Cockell C.S. (2005). Radiative Habitable Zones in Martian Polar Environments. Icarus.

[174] France J.L., King M.D., MacArthur A. (2010). A Photohabitable Zone in the Martian Snowpack? A Laboratory and Radiative-Transfer Study of Dusty Water-ice Snow. Icarus.

[175] ten Kate I.L., Garry J.R.C., Peeters Z., Quinn R., Foing B., Ehrenfreund P. (2005). Amino Acid Photostability on the Martian Surface. Meteorit. Planet. Sci..

[176] Stoker C.R., Bullock M.A. (1997). Organic Degradation under Simulated Martian Conditions. J. Geophys. Res. Planets.

[177] Ten Kate I.L. (2010). Organics on Mars?. Astrobiology.

[178] Oró J., Holzer G. (1979). The Photolytic Degradation and Oxidation of Organic Compounds under Simulated Martian Conditions. J. Mol. Evol..

[179] Scappini F., Casadei F., Zamboni R., Franchi M., Gallori E., Monti S. (2004). Protective Effect of Clay Minerals on Adsorbed Nucleic Acid against UV Radiation: Possible Role in the Origin of Life. Int. J. Astrobiol..

[180] Ciaravella A., Scappini F., Franchi M., Cecchi-Pestellini C., Barbera M., Candia R., Gallori E., Micela G. (2004). Role of Clays in Protecting Adsorbed DNA against X-Ray Radiation. Int. J. Astrobiol..

[181] Garry J.R.C., Ten Kate I.L., Martins Z., Nørnberg P., Ehrenfreund P. (2006). Analysis and Survival of Amino Acids in Martian Regolith Analogs. Meteorit. Planet. Sci..

[182] Biondi E., Branciamore S., Maurel M.-C., Gallori E. (2007). Montmorillonite Protection of an UV-Irradiated Hairpin Ribozyme: Evolution of the RNA World in a Mineral Environment. BMC Evol. Biol..

[183] Shkrob I.A., Chemerisov S.D. (2009). Light Induced Fragmentation of Polyfunctional Carboxylated Compounds on Hydrated Metal Oxide Particles: From Simple Organic Acids to Peptides. J. Phys. Chem. C.

[184] Shkrob I.A., Chemerisov S.D., Marin T.W. (2010). Photocatalytic Decomposition of Carboxylated Molecules on Light-Exposed Martian Regolith and Its Relation to Methane Production on Mars. Astrobiology.

[185] Stalport F., Guan Y.Y., Coll P., Szopa C., Macari F., Raulin F., Chaput D., Cottin H. (2010). UVolution, a Photochemistry Experiment in Low Earth Orbit: Investigation of the Photostability of Carboxylic Acids Exposed to Mars Surface UV Radiation Conditions. Astrobiology.

[186] Johnson A.P., Pratt L.M. (2010). Metal-Catalyzed Degradation and Racemization of Amino Acids in Iron Sulfate Brines under Simulated Martian Surface Conditions. Icarus.

[187] Johnson A.P., Pratt L.M., Vishnivetskaya T., Pfiffner S., Bryan R.A., Dadachova E., Whyte L., Radtke K., Chan E., Tronick S. (2011). Extended Survival of Several Organisms and Amino Acids under Simulated Martian Surface Conditions. Icarus.

[188] Shkrob I.A., Marin T.M., Adhikary A., Sevilla M.D. (2011). Photooxidation of Nucleic Acids on Metal Oxides: Physicochemical and Astrobiological Perspectives. J. Phys. Chem. C.

[189] Dos Santos R., Patel M., Cuadros J., Martins Z. (2016). Influence of Mineralogy on the Preservation of Amino Acids under Simulated Mars Conditions. Icarus.

[190] Ertem G., Ertem M.C., McKay C.P., Hazen R.M. (2017). Shielding Biomolecules from Effects of Radiation by Mars Analogue Minerals and Soils. Int. J. Astrobiol..

[191] Ten Kate I.L., Garry J.R.C., Peeters Z., Foing B., Ehrenfreund P. (2006). The Effects of Martian near Surface Conditions on the Photochemistry of Amino Acids. Planet. Space Sci..

[192] Stalport F., Coll P., Szopa C., Raulin F. (2008). Search for Organic Molecules at the Mars Surface: The “Martian Organic Material Irradiation and Evolution” (MOMIE) Project. Adv. Space Res..

[193] Poch O., Noblet A., Stalport F., Correia J.J., Grand N., Szopa C., Coll P. (2013). Chemical Evolution of Organic Molecules under Mars-like UV Radiation Conditions Simulated in the Laboratory with the “Mars Organic Molecule Irradiation and Evolution” (MOMIE) Setup. Planet. Space Sci..

[194] Onoe J., Kawai T., Kawai S. (1985). Peptide Formation from Amino Acid with Particulate Semiconductor Photocatalysts. Chem. Lett..

[195] Saladino R., Brucato J.R., De Sio A., Botta G., Pace E., Gambicorti L. (2011). Photochemical Synthesis of Citric Acid Cycle Intermediates Based on Titanium Dioxide. Astrobiology.

[196] Li Y., Xu X., Li Y., Ding C., Wu J., Lu A., Ding H., Qin S., Wang C. (2018). Absolute Band Structure Determination on Naturally Occurring Rutile with Complex Chemistry: Implications for Mineral Photocatalysis on Both Earth and Mars. Appl. Surf. Sci..

[197] Ehrenfreund P., Bernstein M.P., Dworkin J.P., Sandford S.A., Allamandola L.J. (2001). The Photostability of Amino Acids in Space. Astrophys. J..

[198] McBride M., Kung K.-H. (1989). Complexation of Glyphosate and Related Ligands with Iron (III). Soil Sci. Soc. Am. J..

[199] Yusenko K., Fox S., Guni P., Strasdeit H. (2008). Model Studies on the Formation and Reactions of Solid Glycine Complexes at the Coasts of a Primordial Salty Ocean. Z. Anorgan. Allg. Chem..

[200] Bada J.L. (1982). Racemization of Amino Acids in Nature. Interdiscip. Sci. Rev..

[201] Kavitha V., Palanivelu K. (2003). Degradation of 2-Chlorophenol by Fenton and Photo-Fenton Processes—A Comparative Study. J. Environ. Sci. Health Part A.

[202] Zuo Y., Hoigne J. (1992). Formation of Hydrogen Peroxide and Depletion of Oxalic Acid in Atmospheric Water by Photolysis of Iron (III)-Oxalato Complexes. Environ. Sci. Technol..

[203] Gu B., Schmitt J., Chen Z., Liang L., McCarthy J.F. (1994). Adsorption and Desorption of Natural Organic Matter on Iron Oxide: Mechanisms and Models. Environ. Sci. Technol..

[204] Williams K.M., Smith G.G. (1977). A Critical Evaluation of the Application of Amino Acid Racemization to Geochronology and Geothermometry. Orig. Life.

[205] Möhlmann D. (2005). Adsorption Water-Related Potential Chemical and Biological Processes in the Upper Martian Surface. Astrobiology.

[206] Franchi M., Gallori E. (2004). Origin, Persistence and Biological Activity of Genetic Material in Prebiotic Habitats. Orig. Life Evol. Biosph..

[207] Otroshchenko V.A., Vasilyeva N.V. (2009). Formation of RNA Oligonucleotides over the Mineral Surface Preliminary Irradiated with UV Light. React. Kinet. Catal. Lett..

[208] Horneck G., Reitz G., Rettberg P., Schuber M., Kochan H., Möhlmann D., Richter L., Seidlitz H. (2000). A Ground-Based Program for Exobiological Experiments on the International Space Station. Planet. Space Sci..

[209] Schuerger A.C., Mancinelli R.L., Kern R.G., Rothschild L.J., McKay C.P. (2003). Survival of Endospores of Bacillus Subtilis on Spacecraft Surfaces under Simulated Martian Environments: Implications for the Forward Contamination of Mars. Icarus.

[210] Schuerger A.C., Richards J.T., Newcombe D.A., Venkateswaran K. (2006). Rapid Inactivation of Seven Bacillus Spp. under Simulated Mars UV Irradiation. Icarus.

[211] Nir E., Kleinermanns K., Grace L., de Vries M.S. (2001). On the Photochemistry of Purine Nucleobases. J. Phys. Chem. A.

[212] Ravanat J.-L., Douki T., Cadet J. (2001). Direct and Indirect Effects of UV Radiation on DNA and Its Components. J. Photochem. Photobiol. B Biol..

[213] Barbatti M., Aquino A.J.A., Szymczak J.J., Nachtigallová D., Hobza P., Lischka H. (2010). Relaxation Mechanisms of UV-Photoexcited DNA and RNA Nucleobases. Proc. Natl. Acad. Sci. USA.

[214] Pavlov A.K., Blinov A.V., Konstantinov A.N. (2002). Sterilization of Martian Surface by Cosmic Radiation. Planet. Space Sci..

[215] Clark B.C. (1979). Chemical and Physical Microenvironments at the Viking Landing Sites. J. Mol. Evol..

[216] Gerakines P.A., Hudson R.L., Moore M.H., Bell J.-L. (2012). In Situ Measurements of the Radiation Stability of Amino Acids at 15–140 K. Icarus.

[217] López-Esquivel Kranksith L., Negrón-Mendoza A., Mosqueira F.G., Ramos-Bernal S. (2010). Radiation-Induced Reactions of Amino Acids Adsorbed on Solid Surfaces. Nucl. Instrum. Methods Phys. Res. Sect. A.

[218] Guzmán A., Negrón-Mendoza A., Ramos-Bernal S. (2002). Behavior of Adenine in Na-Montmorillonite Exposed to Gamma Radiation: Implications to Chemical Evolution Studies. Cell. Mol. Biol..

[219] Góbi S., Förstel M., Maksyutenko P., Kaiser R.I. (2017). A Reflectron Time-of-Flight Mass Spectrometric Study on the Degradation Pathways of Glycine on Mars in the Presence of Perchlorates and Ionizing Radiation. Astrophys. J..

[220] Schoonen M., Smirnov A., Cohn C. (2004). A Perspective on the Role of Minerals in Prebiotic Synthesis. AMBIO J. Hum. Environ..

[221] Gallori E., Biondi E., Franchi M., Seckbach J., Chela-Flores J., Owen T., Raulin F. (2004). Mineral Surfaces as a Cradle of Primordial Genetic Material. Life in the Universe.

[222] Keppler F., Vigano I., McLeod A., Ott U., Früchtl M., Röckmann T. (2012). Ultraviolet-Radiation-Induced Methane Emissions from Meteorites and the Martian Atmosphere. Nature.

[223] Okabe H. (1978). Photochemistry of Small Molecules.

[224] Shemansky D.E. (1972). CO_2_ Extinction Coefficient 1700–3000 Å. J. Chem. Phys..

[225] Karaiskou A., Vallance C., Papadakis V., Vardavas I.M., Rakitzis T.P. (2004). Absolute Absorption Cross-Section Measurements of CO_2_ in the Ultraviolet from 200 to 206 Nm at 295 and 373 K. Chem. Phys. Lett..

[226] Vesela A., Wilhelm J. (2002). The Role of Carbon Dioxide in Free Radical Reactions in Organism. Physiol. Res..

[227] Ehlmann B.L., Mustard J.F. (2012). An In-Situ Record of Major Environmental Transitions on Early Mars at Northeast Syrtis Major. Geophys. Res. Lett..

[228] Goudge T.A., Mustard J.F., Head J.W., Fassett C.I., Wiseman S.M. (2015). Assessing the Mineralogy of the Watershed and Fan Deposits of the Jezero Crater Paleolake System, Mars. J. Geophys. Res. Planets.

[229] Salvatore M.R., Goudge T.A., Bramble M.S., Edwards C.S., Bandfield J.L., Amador E.S., Mustard J.F., Christensen P.R. (2018). Bulk Mineralogy of the NE Syrtis and Jezero Crater Regions of Mars Derived through Thermal Infrared Spectral Analyses. Icarus.

[230] Bridges J.C., Loizeau D., Sefton-Nash E., Vago J., Williams R.M.E., Balme M., Turner S.M.R., Fawdon P., Davis J.M. Selection and Characterization of the ExoMars 2020 Rover Landing Sites. Proceedings of the 48th Lunar and Planetary Science Conference.

[231] Amiri S., McGrath M., Al Awadhi M., Almatroushi H., Sharaf O., AlDhafri S., AlRais A., Wali M., AlShamsi Z., AlQasim I. Emirates Mars Mission (EMM) 2020 Overview. Proceedings of the 42nd COSPAR Scientific Assembly.

[232] Jiang X., Yang B., Li S. (2018). Overview of China’s 2020 Mars Mission Design and Navigation. Astrodynamics.

[233] Haider S.A., Bhardwaj A., Shanmugam M., Goyal S.K., Sheel V., Pabari J., Prasad Karanam D. Indian Mars and Venus Missions: Science and Exploration. Proceedings of the 42nd COSPAR Scientific Assembly.

[234] Campagnola S., Yam C.H., Tsuda Y., Ogawa N., Kawakatsu Y. (2018). Mission Analysis for the Martian Moons Explorer (MMX) Mission. Acta Astronaut..

